# Anion-templated silver nanoclusters: precise synthesis and geometric structure

**DOI:** 10.1080/14686996.2023.2203832

**Published:** 2023-05-25

**Authors:** Yusuke Horita, Mai Ishimi, Yuichi Negishi

**Affiliations:** a Department of Applied Chemistry, Faculty of Science, Tokyo University of Science, Shinjuku-ku, Japan; b Research Institute for Science & Technology, Tokyo University of Science, Shinjuku-ku, Japan

**Keywords:** Metal cluster, silver cluster, anion-templated cluster, geometric structure, synthesis method

## Abstract

Metal nanoclusters (NCs) are gaining much attention in nanoscale materials research because they exhibit size-specific physicochemical properties that are not observed in the corresponding bulk metals. Among them, silver (Ag) NCs can be precisely synthesized not only as pure Ag NCs but also as anion-templated Ag NCs. For anion-templated Ag NCs, we can expect the following capabilities: 1) size and shape control by regulating the central anion (anion template); 2) stabilization by adjusting the charge interaction between the central anion and surrounding Ag atoms; and 3) functionalization by selecting the type of central anion. In this review, we summarize the synthesis methods and influences of the central anion on the geometric structure of anion-templated Ag NCs, which include halide ions, chalcogenide ions, oxoanions, polyoxometalate, or hydride/deuteride as the central anion. This summary provides a reference for the current state of anion-templated Ag NCs, which may promote the development of anion-templated Ag NCs with novel geometric structures and physicochemical properties.

## GRAPHICAL ABSTRACT

**Figure:**
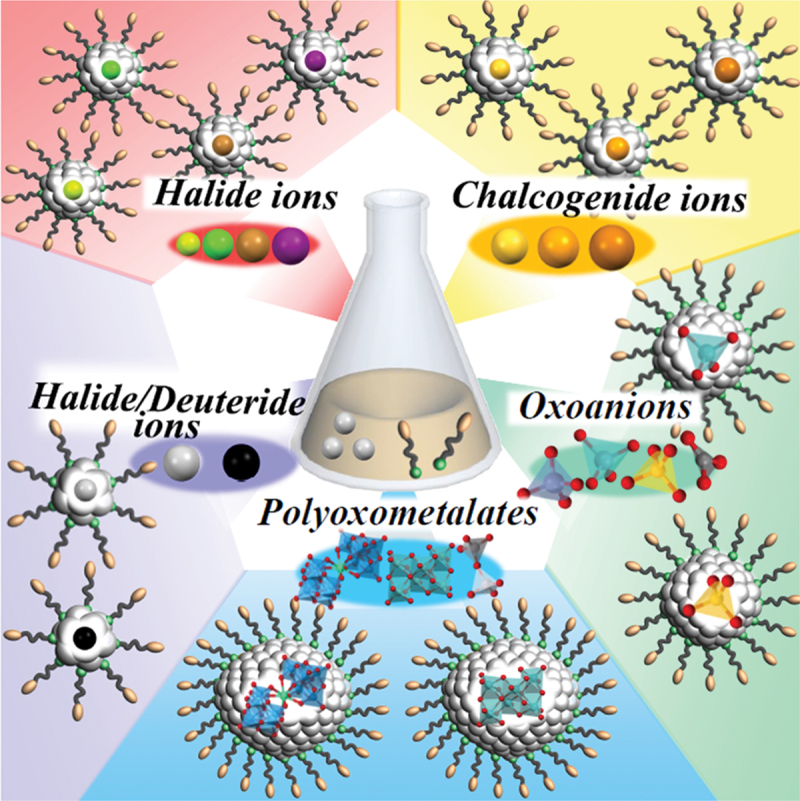

## 1. Introduction

Aggregates of a few to several hundred metal atoms are called metal nanoclusters (NCs). Metal NCs are very small, with a particle size of 1–2 nm, which can give rise to novel geometric structures and discretization of electronic levels [1–13]. Related to these factors, size-specific physicochemical properties can emerge in metal NCs that are not observed in the corresponding bulk metals [14–17]. Accordingly, metal NCs have attracted much attention in academic fields and industries related to materials chemistry [18–22].

Among the metal NCs, those composed of coinage metals, such as gold (Au) [23–85], silver (Ag) [86–103], and copper (Cu) [104–107], are particularly promising as novel functional materials because of their high stability. In particular, Ag NCs have been increasingly studied in recent years because of their luminescence with high quantum yield [108] and the specific catalytic ability for carbon dioxide (CO_2_) reduction [109]. These Ag NCs can be synthesized with atomical precision using ligands, such as thiolate (SR) [110–115], phosphine (PR_3_) [116–118], and acetylide (C≡CR) [119,120]. For ligand-protected Ag NCs containing anions at the central position (hereafter, anion-templated Ag NCs), synthesis of new NCs has been continuously reported since 1996.

**Figure 1.**
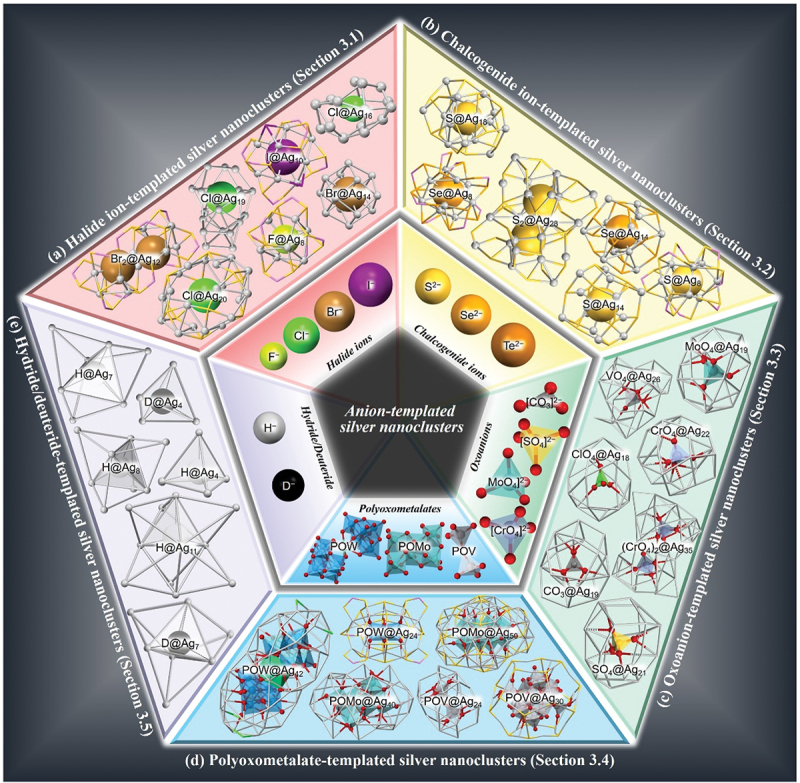
Diversification of anion-templated Ag NCs. (a) X-templated Ag NCs (X@Ag NCs; Section 3.1), (b) X’-templated Ag NCs (X’@Ag NCs; Section 3.2), (c) Ox-templated Ag NCs (Ox@Ag NCs; Section 3.3), (d) POM-templated Ag NCs (Pom@Ag NCs; Section 3.4), and (e) H/D-templated Ag NCs (H/D@Ag NCs; Section 3.5).

The anion-templated metal NCs were discovered in the synthesis of Cu NCs. In 1976, Freeman et al. [121] and Schugar et al. [122] both synthesized Cl@Cu_14_Cl(SC(CH_3_)_2_CH_2_NH_2_)_12_, where SC(CH_3_)_2_CH_2_NH_2_ is penicillamine, and the element or compound before ‘@’ is the central anion. In this case, chloride ions (Cl^–^), which were included in the reagents, served as the template anion. In 1993, Tang and colleagues reported on S@Cu_14_(SPh)_12_(PPh_3_)_6_ (S = sulfide ion, SPh = benzenethiolate, PPh_3_ = triphenylphosphine, Table 1) [123], where carbon disulfide (CS_2_) was used as the source of a template anion (S^2−^), and thus, anion-templated Cu NCs were strategically synthesized. Later, they successfully determined the geometric structure of S@Ag_14_(SPh)_12_(PPh_3_)_8_·4MeOH·13H_2_O (MeOH = methanol, H_2_O = water) [124], which is the first report on anion-templated Ag NCs. In 2009, Wang and colleagues reported the synthesis of [CO_3_@Ag_17_(C≡C^*t*^Bu)_14_]^+^ (CO_3_ = carbonate ion, C≡C^*t*^Bu = *tert*-butylacetylene, Table 1) [125], which demonstrated that oxoanions (Ox) can also function as a template. Recently, Ag NCs encapsulating polyoxometalate (POM), which is formed by the condensation of Ox, hydride (H), or deuteride (D), have also been reported, and the types of anion species in anion-templated Ag NCs are diversifying (Figure 1).

**Table 1.** The abbreviations for functional group names and compound names.

Abbreviation	Full name	Chemical formula
Ar	4-Anisyl group	–C_6_H_4_OCH_3_
^*s*^Bu	*sec*-Butyl group	–CH(CH_3_)CH_2_CH_3_
^*t*^Bu	*tert*-Butyl group	–C(CH_3_)_3_
Bz	Benzyl group	–CH_2_C_6_H_5_
Cy	Cyclohexyl group	–C_6_H_11_
Et	Ethyl group	–CH_2_CH_3_
Fc	Ferrocenyl group	C_5_H_5_FeC_5_H_4_–
Me	Methyl group	–CH_3_
Nap	Naphthalene group	–C_10_H_7_
Ph	Phenyl group	–C_6_H_5_
	Phenylene group	–C_6_H_4_–
Pr	Propyl group	–CH_2_CH_2_CH_3_
^*i*^Pr	*iso*-Propyl group	–CH(CH_3_)CH_3_
*p*-tol	*p*-Tolyl group	–C_6_H_4_CH_3_
*p*-tos	Tosyl group	–SO_2_C_6_H_4_CH_3_
OAc	Acetate	CH_3_CO_2_
S-Adm	1-Adamantanethioate	SC_10_H_16_
Ala	Alanine	CH_3_CH(NH_2_)CO_2_H
BMIm	1-Butyl-3-methylimidazolium	CH_3_(CH_2_)_3_NC_3_H_3_NCH_3_
4Cp	4-Cyanopyridine	NC_5_H_4_CN
Cys	Cysteine	HSCH_2_CH(NH_2_)CO_2_H
DCM	Dichloromethane	CH_2_Cl_2_
DEF	Diethyl formamide	(C_2_H_5_)_2_NCHO
DMF	*N,N*-Dimethylformamide	(CH_3_)_2_NCHO
DMAc	Dimethylacetamide	CH_3_C(O)N(CH_3_)_2_
dppb	1,4-Bis(diphenylphosphino)butane	(C_6_H_5_)_2_PC_4_H_8_P(C_6_H_5_)_2_
dppe	1,2-Bis(diphenylphosphino)ethane	(C_6_H_5_)_2_PC_2_H_4_P(C_6_H_5_)_2_
dppf	1,1′-Bis(diphenylphosphino)ferrocene	(C_6_H_5_)_2_P[Fe(C_5_H_4_)_2_]P(C_6_H_5_)_2_
dppm	Bis(diphenylphosphino)methane	(C_6_H_5_)_2_PCH_2_P(C_6_H_5_)_2_
dppp	1,3-Bis(diphenylphosphino)propane	(C_6_H_5_)_2_PC_3_H_6_P(C_6_H_5_)_2_
hfac	Hexafluoroacetylacetone	CF_3_C(O)CC(O)CF_3_
OTf	(Trifluoromethyl)sulfonate	CF_3_SO_3_
tfa	Trifluoroacetate	CF_3_CO_2_
THF	Tetrahydrofuran	C_4_H_8_O
TMEDA	*N,N,N′,N′*-Tetramethylethylenediamine	(CH_3_)_2_NC_2_H_4_N(CH_3_)_2_
H_2_PTA	Phthalic acid	C_6_H_4_(CO_2_H)_2_
Pro	Proline	C_4_H_7_NHCO_2_H
Py	Pyridine	C_5_H_5_N
Val	Valine	(CH_3_)_2_CHCH(NH_2_)CO_2_H

In the synthesis of Ag NCs, the anion-template method is effective for generating new structures and new functions. Specifically, 1) the size and shape of Ag NCs can be controlled, 2) the charge interactions within Ag NCs can be well stabilized, and 3) novel functions can be added to Ag NCs, all depending on the selection of the central anion. Notably, several reviews have focused on anion-templated Ag NCs [126–130], but these reviews are limited and do not comprehensively summarize the synthesis methods and geometric structures obtained using all of the aforementioned anions (halide (X), chalcogenide (X’), Ox, POM, and H/D).

In this review, we categorize anions into five types, namely X, X’, Ox, POM, and H/D, and describe the synthesis and geometric structures of anion-templated Ag NCs encapsulating these anions. The paper discusses the influences of central anions on the geometric structure of anion-templated Ag NCs by comparing representative anion-templated Ag NCs. Owing to space limitations, all the chemical compositions of the reported anion-templated Ag NCs that are not described in detail are summarized in Tables 2–6. This summary aims to provide readers with the latest knowledge on anion-templated Ag NCs, serving as design guidelines for the creation of novel structures.

**Table 2.** Representative X-templated Ag NCs.

No.	Chemical formula	Abbreviation	Center anion	*z*^*a*^	Synthesis method	Year	Ref.
1	[Br@Ag_7_(dppe)_3_(SeC_2_H_4_O(O)CFc)_6_]^0^	**Br@Ag7**	Br^–^	0	Stirring	2011	[156]
2	[F@Ag_8_{S_2_P(OEt)_2_}_6_][PF_6_]	**F@Ag8a**	F^–^	+1	Stirring	2010	[132]
3	[F@Ag_8_{S_2_P(O^*i*^Pr)_2_}_6_][PF_6_]	**F@Ag8b**	F^–^	+1	Stirring	2014	[133]
4	[Cl@Ag_8_{Se_2_P(OEt)_2_}_6_][PF_6_]	**Cl@Ag8a**	Cl^–^	+1	Stirring	2004	[131]
5	[Cl@Ag_8_{Se_2_P(OPr)_2_}_6_][PF_6_]	**Cl@Ag8b**	Cl^–^	+1	Stirring	2004	[131]
6	[Cl@Ag_8_{Se_2_P(O^*i*^Pr)_2_}_6_][PF_6_]	**Cl@Ag8c**	Cl^–^	+1	Stirring	2004	[131]
7	[Cl@Ag_8_{S_2_P(OEt)_2_}_6_][PF_6_]	**Cl@Ag8d**	Cl^–^	+1	Stirring	2010	[132]
8	[Cl@Ag_8_{S_2_P(O^*i*^Pr)_2_}_6_][PF_6_]	**Cl@Ag8e**	Cl^–^	+1	Stirring	2014	[133]
9	[Br@Ag_8_{Se_2_P(OEt)_2_}_6_][PF_6_]	**Br@Ag8a**	Br^–^	+1	Stirring	2004	[131]
10	[Br@Ag_8_{Se_2_P(OPr)_2_}_6_][PF_6_]	**Br@Ag8b**	Br^–^	+1	Stirring	2004	[131]
11	[Br@Ag_8_{Se_2_P(O^*i*^Pr)_2_}_6_][PF_6_]	**Br@Ag8c**	Br^–^	+1	Stirring	2004	[131]
12	[Br@Ag_8_{S_2_P(O^*i*^Pr)_2_}_6_][PF_6_]	**Br@Ag8d**	Br^–^	+1	Stirring	2014	[133]
13	[I@Ag_10_I_3_{S_2_P(O^*i*^Pr)_2_}_6_]^0^	**I@Ag10**	I^–^	0	Stirring	2014	[133]
14	[I@Ag_11_I_3_{S_2_P(O^*i*^Pr)_2_}_6_][PF_6_]	**I@Ag11a**	I^–^	+1	Stirring	2012	[157]
15	[I@Ag_11_I_3_{Se_2_P(O^*i*^Pr)_2_}_6_][PF_6_]	**I@Ag11b**	I^–^	+1	Stirring	2012	[157]
16	[Br_2_@Ag_12_{S_2_P(OEt)_2_}_10_]^0^	**Br**_**2**_**@Ag12**	Br^–^	0	Stirring	2013	[146]
17	[I@Ag_12_I_4_{S_2_P(C_2_H_4_Ph)_2_}_6_][I]	**I@Ag12**	I^–^	+1	Stirring	2014	[145]
18	[I_2_@Ag_12_{S_2_P(OEt)_2_}_10_]^0^	**I**_**2**_**@Ag12a**	I^–^	0	Stirring	2013	[146]
19	[I_2_@Ag_12_{Se_2_P(OEt)_2_}_10_]^0^	**I**_**2**_**@Ag12b**	I^–^	0	Stirring	2013	[146]
20	[F@Ag_14_(C≡C^*t*^Bu)_12_][BF_4_]	**F@Ag14**	F^–^	+1	Stirring	2002	[148]
21	[Cl@Ag_14_(C≡C^*t*^Bu)_12_][OH]	**Cl@Ag14a**	Cl^–^	+1	Treating^*b*^	2001	[147]
22	[Cl@Ag_14_(C≡C^*t*^Bu)_12_][BF_4_]	**Cl@Ag14b**	Cl^–^	+1	Stirring	2001	[147]
23	[Br@Ag_14_(C≡C^*t*^Bu)_12_][BF_4_]	**Br@Ag14**	Br^–^	+1	Stirring	2001	[147]
24	[Cl@Ag_8_Ni_6_{SCMe_2_CH(NH_2_)CO_2_}_12_]_3_ [Co(NH_3_)_6_]_5_·~197 H_2_O	**Cl@Ag8Ni6**	Cl^–^	–5	Crystallization^*b*^	1981	[160]
25	[Cl@Ag_8_Cu_6_(C≡C^*t*^Bu)_12_][BF_4_]	**Cl@Ag8Cu6**	Cl^–^	+1	Stirring	2013	[161]
26	[Br@Ag_8_Cu_6_(C≡C^*t*^Bu)_12_][BF_4_]	**Br@Ag8Cu6**	Br^–^	+1	Stirring	2013	[161]
27	[Cl@Ag_16_(C≡C^*t*^Bu)_9_(hfac)(OAc)] [Yb_3_OH_4_(^*t*^BuPO_3_)_3_(hfac)_3_(MeOH)_3_]^0^·4MeOH·H_2_O	**Cl@Ag16a**	Cl^–^	0	Stirring	2018	[158]
28	[Cl@Ag_16_(C≡C^*t*^Bu)_7_(hfac)_2_(^*t*^BuPO_3_)(MeOH)]_2_[Yb_6_(OH)_2_O_6_(^*t*^BuPO_3_)_6_(H_2_O)_6_(MeOH)_6_]^0^	**Cl@Ag16b**	Cl^–^	0	Stirring	2018	[158]
29	[Cl@Ag_16_(C≡C^*t*^Bu)_11_(hfac)_3_(tfa)]^0^	**Cl@Ag16c**	Cl^–^	0	Ultrasonication	2018	[159]
30	[Cl@Ag_16_(S^*t*^Bu)_8_(tfa)_7_(DMF)_4_(H_2_O)]^0^·1.5DMF	**Cl@Ag16d**	Cl^–^	0	Stirring	2019	[162]
31	[Cl@Ag_16_S(S-Adm)_8_(tfa)_5_(DMF)_3_(H_2_O)_2_]^0^·DMF	**Cl@Ag16e**	Cl^–^	0	Stirring	2022	[163]
32	[Cl@Ag_12_(C≡CPh)_14_(AgPPh_3_)_6_][SbF_6_]_4_[CH_2_ClNEt_3_]	**Cl@Ag18a**	Cl^–^	+3	Stirring	2017	[149]
33	[Cl@Ag_12_(C≡CPh)_14_{AgP(*p*-tol)_3_}_6_][SbF_6_]_3_	**Cl@Ag18b**	Cl^–^	+3	Stirring	2017	[149]
34	[Cl@Ag_19_(C≡C^*t*^Bu)_11_(tfa)_7_]^0^	**Cl@Ag19**	Cl^–^	0	Stirring	2008	[150]
35	[Cl@Ag_20_(S^*t*^Bu)_10_(tfa)_2_][tfa]_7_·5MeOH	**Cl@Ag20**	Cl^–^	+7	Stirring	2013	[151]
36	[Cl_2_@Ag_21_(C≡C^*t*^Bu)_9_{(^*t*^BuPO_3_)_3_V_3_O_6_(OH)}_2_{(^*t*^BuPO_3_)VO_2_(OH)}(MeOH)_2_(H_2_O)_2_]^0^·2MeOH·2H_2_O	**Cl**_**2**_**@Ag21**	Cl^–^	0	Stirring	2012	[152]
37	[Br@Ag_36_(SPh^*t*^Bu)_36_][Et_3_NH]	**Br@Ag36**	Br^–^	−1	Solvothermal	2010	[153]
38	[Cl@Ag_12_@Ag_48_(dppm)_12_]^0^	**Cl@Ag60**	Cl^–^	0	Crystallization^*b*^	2019	[154]
39	[Cl@Ag_216_S_56_Cl_6_(C≡CPh)_98_(H_2_O)_12_] [Ag_3_(C_3_H_4_N_2_)(H_2_O)_4_][SbF_6_]_2_	**Cl@Ag216**	Cl^–^	+1	Stirring	2017	[155]

^*a*^Charge state of Ag NC. ^*b*^See references for details of the synthesis method.

**Table 3.** Representative X’-templated Ag NCs.

No.	Chemical formula	Abbreviation	Center anion	*z*	Synthesis method	Year	Ref.
40	[S@Ag_8_{S_2_P(O^*i*^Pr)_2_}_6_]^0^	**S@Ag8**	S^2–^	0	Stirring	2014	[133]
41	[Se@Ag_8_{Se_2_P(O^*i*^Pr)_2_}_6_]^0^	**Se@Ag8**	Se^2–^	0	Stirring	1999	[179]
42	[S@Ag_9_{S_2_P(OEt)_2_}_8_][Na]	**S@Ag9**	S^2–^	−1	Stirring	2017	[134]
43	[Se@Ag_9_{Se_2_P(OEt)_2_}_8_][Na]	**Se@Ag9**	Se^2–^	−1	Stirring	2017	[134]
44	[S@Ag_10_{S_2_P(OEt)_2_}_8_]^0^	**S@Ag10**	S^2–^	0	Stirring	2004	[169]
45	[Se@Ag_10_{Se_2_P(OEt)_2_}_8_]^0^	**Se@Ag10a**	Se^2–^	0	Stirring	2002	[168]
46	[Se@Ag_10_{Se_2_P(O^*i*^Pr)_2_}_8_]^0^	**Se@Ag10b**	Se^2–^	0	Stirring	2002	[168]
47	[S@Ag_11_{S_2_P(OEt)_2_}_8_][PF_6_]	**S@Ag11a**	S^2–^	+1	Stirring	2011	[170]
48	[S@Ag_11_{S_2_P(OPr)_2_}_8_][PF_6_]	**S@Ag11b**	S^2–^	+1	Stirring	2011	[170]
49	[S@Ag_11_{S_2_P(O^*i*^Pr)_2_}_8_][PF_6_]	**S@Ag11c**	S^2–^	+1	Stirring	2011	[170]
50	[S@Ag_11_(C≡C^*t*^Bu)_2_{S_2_P(OEt)_2_}_7_]^0^	**S@Ag11d**	S^2–^	0	Stirring	2018	[177]
51	[Se@Ag_11_I_3_{Se_2_P(OEt)_2_}_6_]^0^	**Se@Ag11a**	Se^2–^	0	Stirring	2006	[178]
52	[Se@Ag_11_I_3_{Se_2_P(O^*i*^Pr)_2_}_6_]^0^	**Se@Ag11b**	Se^2–^	0	Stirring	2006	[178]
53	[Se@Ag_11_I_3_{Se_2_P(O^*s*^Bu)_2_}_6_]^0^	**Se@Ag11c**	Se^2–^	0	Stirring	2006	[178]
54	[S@Ag_14_(SPh)_12_(PPh_3_)_8_]^0^·4MeOH·13H_2_O	**S@Ag14a**	S^2–^	0	Stirring	1996	[124]
55	[S@Ag_14_(SPhCN)_12_(PPh_3_)_8_][FeCl_2_(THF)_4_]_3_	**S@Ag14b**	S^2–^	0	Stirring	2011	[174]
56	[S@Ag_14_(SPhCNS)_12_(PPh_3_)_8_]^0^	**S@Ag14c**	S^2–^	0	Treating^*a*^	2011	[174]
57	[S@Ag_14_(PPh_3_)_6_(SC_2_H_4_O(O)CFc)_12_]^0^	**S@Ag14d**	S^2–^	0	Stirring	2011	[156]
58	[S@Ag_14_(C≡CPh)_8_{S_2_P(OEt)_2_}_4_(TMEDA)_2_]^0^·5MeOH	**S@Ag14e**	S^2–^	0	Stirring	2018	[177]
59	[S@Ag_14_(C≡CPh)_8_{S_2_P(O^*i*^Pr)_2_}_4_(TMEDA)_2_]^0^·7MeOH	**S@Ag14f**	S^2–^	0	Stirring	2018	[177]
60	[Se@Ag_6_(AgPPh_3_)_8_(SePh)_12_]^0^·11THF	**Se@Ag14**	Se^2–^	0	Treating^*a*^	2010	[175]
61	[Se_0.5_@Ag_6_(AgPPh_3_)_8_(SePh)_12_][Ph_3_SnCl_2_]·6THF	**Se**_**0.5**_**@Ag14a**	Se^2–^	+1	Treating^*a*^	2010	[175]
62	[Se_0.5_@Ag_6_(AgPPh_3_)_8_(SePh)_12_][Cy_3_SnCl_2_]·5THF	**Se**_**0.5**_**@Ag14b**	Se^2–^	+1	Treating^*a*^	2010	[175]
63	[S@Ag_15_(S^*s*^Bu)_12_(dppb)_3_][OTf]·H_2_O	**S@Ag15**	S^2–^	+1	Solvothermal	2021	[180]
64	[S@Ag_17_(SPhCO_2_H)_16_][NH_4_]_17_·22H_2_O	**S@Ag17**	S^2–^	−17	Ultrasonication	2011	[144]
65	[S@Ag_18_(SPh^*t*^Bu)_16_(dppp)_4_]^0^ ·DMF·5MeCN·3MeOH	**S@Ag18a**	S^2–^	0	Solvothermal	2017	[135]
66	[S@Ag_18_(SCH_2_Ph^*t*^Bu)_16_(PPh_3_)_8_]^0^	**S@Ag18b**	S^2–^	0	Stirring	2020	[171]
67	[S@Ag_20_(S^*t*^Bu)_10_(tfa)_8_(MeCN)_4_]^0^·EtOH	**S@Ag20**	S^2–^	0	Treating^*a*^	2022	[181]
68	[S_2_@Ag_23_(SPhOSiMe_3_)_15_(SPhOH)_3_(PPh_3_)_8_]^0^	**S**_**2**_**@Ag23**	S^2–^	0	Stirring	2011	[174]
69	[S_2_@Ag_28_(ArP(O)S_2_)_12_(PPh_3_)_12_]^0^	**S**_**2**_**@Ag28**	S^2–^	0	Stirring	2005	[176]
70	[[S@Ag_12_@(^*n*^BuPO_3_)_9_@Ag_36_(S^*t*^Bu)_23_ (CH_3_O)_2_(NO_3_)_3_]^0^·2MeOH]_*n*_	**[S@Ag48]**_***n***_	S^2–^	0	Stirring	2015	[172]
71	[S@Ag_50_S_12_(S^*t*^Bu)_20_][tfa]_4_	**S@Ag50**	S^2–^	+4	Crystallization	2022	[182]
72	[[S@Ag_11_@(^*n*^BuPO_3_)_7_(MoO_4_)_2_@Ag_40_(S^*t*^Bu)_27_(CH_3_O)_2_(NO_3_)_2_(H_2_O)_2_]^0^·8MeOH·1.5H_2_O]_*n*_	**[S@Ag51]**_***n***_	S^2–^	0	Stirring	2015	[172]
73	[S@Ag_56_S_12_(S^*t*^Bu)_20_]·10OAc·6DMF·MeOH	**S@Ag56**	S^2–^	+10	Ultrasonication	2013	[143]
74	[S@Ag_60_S_14_(S^*i*^Pr)_24_(OTf)_14_(MeOH) (DMF)_2_·2DCM]^0^·8H_2_O	**S@Ag60**	S^2–^	0	Solvothermal	2018	[137]
75	[S@Ag_62_S_12_(S^*t*^Bu)_32_][BF_4_]_4_	**S@Ag62**	S^2–^	+4	Solvothermal	2010	[173]

^*a*^See citation for detailed synthesis method.

**Table 4.** Representative Ox-templated Ag NCs.

No.	Chemical formula	Abbreviation	Center anion	*z*	Synthesis method	Year	Ref.
76	[CO_3_@Ag_12_(C≡C^*t*^Bu)_4_{S_2_P(OEt)_2_}_6_]^0^·0.5H_2_O	**CO**_**3**_**@Ag12**	[CO_3_]^2–^	0	Stirring	2018	[177]
77	[NO_3_@Ag_15_(*o*-FPhC≡C)_10_][NO_3_]_4_	**NO**_**3**_**@Ag15**	[NO_3_]^–^	+4	Stirring	2014	[185]
78	[(NO_3_)_2_@Ag_16_(C≡CPh)_4_[(^*t*^BuPO_3_)_4_V_4_O_8_]_2_ (DMF)_6_(NO_3_)_2_]^0^·DMF·H_2_O	**(NO**_**3**_**)**_**2**_**@Ag16a**	[NO_3_]^–^	0	Stirring	2011	[184]
79	[(NO_3_)_2_@Ag_16_(C≡CPh)_4_{(^*t*^BuPO_3_)_4_V_4_O_8_}_2_(DEF)_6_ (NO_3_)_2_]^0^	**(NO**_**3**_**)**_**2**_**@Ag16b**	[NO_3_]^–^	0	Stirring	2012	[198]
80	[(NO_3_)_2_@Ag_16_(C≡C^*t*^Bu)_4_{(^*t*^BuPO_3_)_4_V_4_O_8_}_2_(DMF)_6_ (NO_3_)_2_]^0^·DMF·2H_2_O	**(NO**_**3**_**)**_**2**_**@Ag16c**	[NO_3_]^–^	0	Stirring	2012	[198]
81	[(NO_3_)_2_@Ag_16_(C≡C^*t*^Bu)_4_{(^*t*^BuPO_3_)_4_V_4_O_8_}_2_(DMF)_6_ (NO_3_)_2_]·[(NO_3_)_2_@Ag_16_(C≡C^*t*^Bu)_4_{(^*t*^BuPO_3_)_4_V_4_O_8_}_2_ (DMF)_4_(Py)_2_(NO_3_)_2_]·DMF·5H_2_O	**[(NO**_**3**_**)**_**2**_ **@Ag16]**_**2**_	[NO_3_]^–^	0	Stirring	2012	[198]
82	[SO_3_@Ag_16_{S_2_P(OEt)_2_}_12_]_2_[PF_6_]_4_	**[SO**_**3**_**@Ag16]**_**2**_	[SO_3_]^2–^	+4	Stirring	2014	[192]
83	[SeO_3_@Ag_16_{S_2_P(OEt)_2_}_12_]_2_[PF_6_]_4_	**[SeO**_**3**_**@Ag16]**_**2**_	[SeO_3_]^2–^	+4	Stirring	2014	[192]
84	[TeO_3_@Ag_16_{S_2_P(OEt)_2_}_12_]_2_[PF_6_]_4_	**[TeO**_**3**_**@Ag16]**_**2**_	[TeO_3_]^2–^	+4	Stirring	2014	[192]
85	[C_2_O_4_@Ag_16_{S_2_P(OEt)_2_}_12_]_2_[PF_6_]_4_	**[C**_**2**_**O**_**4**_**@Ag16]**_**2**_	[C_2_O_4_]^2–^	+4	Stirring	2022	[201]
86	[C_4_O_4_@Ag_16_{S_2_P(OEt)_2_}_12_]_2_[PF_6_]_4_	**[C**_**4**_**O**_**4**_**@Ag16]**_**2**_	[C_4_O_4_]^2–^	+4	Stirring	2022	[201]
87	[SO_4_@Ag_16_{S_2_P(OEt)_2_}_12_]_2_[PF_6_]_4_	**[SO**_**4**_**@Ag16]**_**2**_	[SO_4_]^2–^	+4	Stirring	2011	[191]
88	[SeO_4_@Ag_16_{S_2_P(OEt)_2_}_12_]_2_[PF_6_]_4_	**[SeO**_**4**_**@Ag16]**_**2**_	[SeO_4_]^2–^	+4	Stirring	2011	[191]
89	[CrO_4_@Ag_16_{S_2_P(OEt)_2_}_12_]_2_[PF_6_]_4_	**[CrO**_**4**_**@Ag16]**_**2**_	[CrO_4_]^2–^	+4	Stirring	2011	[191]
90	[MoO_4_@Ag_16_{S_2_P(OEt)_2_}_12_]_2_[PF_6_]_4_	**[MoO**_**4**_**@Ag16]**_**2**_	[MoO_4_]^2–^	+4	Stirring	2011	[191]
91	[CO_3_@Ag_17_(C≡C^*t*^Bu)_14_][OTf]	**CO**_**3**_**@Ag17**	[CO_3_]^2–^	+1	Stirring	2009	[125]
92	[ClO_4_@Ag_18_(C≡C^*i*^Pr)_12_][ClO_4_]_5_	**ClO**_**4**_**@Ag18**	[ClO_4_]^–^	+5	Stirring	2014	[185]
93	[CrO_4_@Ag_18_(C≡C^*i*^Pr)_12_][ClO_4_]_4_	**CrO**_**4**_**@Ag18**	[CrO_4_]^2–^	+4	Stirring	2014	[185]
94	[CO_3_@Ag_19_(C≡C^*t*^Bu)_16_][BF_4_]·MeOH	**CO**_**3**_**@Ag19**	[CO_3_]^2–^	+1	Stirring	2009	[125]
95	[NO_3_@Ag_19_(S^*t*^Bu)_10_(tfa)_8_(4Cp)]^0^·2H_2_O	**NO**_**3**_**@Ag19**	[NO_3_]^–^	0	Ultrasonication	2022	[204]
96	[SO_4_@Ag_19_(C≡C^*t*^Bu)_8_ (Ph_2_PO_2_)_6_(tfa)_3_(MeOH)_2_]^0^	**SO**_**4**_**@Ag19**	[SO_4_]^2–^	0	Solvothermal	2022	[203]
97	[CrO_4_@Ag_19_(C≡C^*t*^Bu)_8_ (Ph_2_PO_2_)_6_(tfa)_3_(MeOH)_2_]^0^	**CrO**_**4**_**@Ag19**	[CrO_4_]^2–^	0	Solvothermal	2022	[203]
98	[MoO_4_@Ag_19_(C≡C^*t*^Bu)_12_(hfac)_2_(tfa)_3_]^0^	**MoO**_**4**_**@Ag19**	[MoO_4_]^2–^	0	Ultrasonication	2018	[159]
99	[CO_3_@Ag_20_(S^*t*^Bu)_10_(DMF)_6_(NO_3_)_8_]^0^	**CO**_**3**_**@Ag20a**	[CO_3_]^2–^	0	Ultrasonication	2013	[141]
100	[CO_3_@Ag_20_(S^*t*^Bu)_10_(OAc)_8_(DMF)_4_]^0^ ·DMF·MeOH	**CO**_**3**_**@Ag20b**	[CO_3_]^2–^	0	Ultrasonication	2013	[143]
101	[CO_3_@Ag_20_(S^*t*^Bu)_10_(NO_3_)_8_(DMAc)_4_]^0^	**CO**_**3**_**@Ag20c**	[CO_3_]^2–^	0	Ultrasonication	2018	[206]
102	[CO_3_@Ag_20_(S^*t*^Bu)_10_(PhCO_2_)_8_(DMAc)_2_]^0^·2MeCN	**CO**_**3**_**@Ag20d**	[CO_3_]^2–^	0	Stirring	2018	[206]
103	[CO_3_@Ag_20_(S^*t*^Bu)_10_(C_12_H_6_O_2_NCH_2_CO_2_)_8_(MeCN)_2_]^0^·2MeCN	**CO**_**3**_**@Ag20e**	[CO_3_]^2–^	0	Stirring	2018	[206]
104	[CO_3_@Ag_20_(S^*t*^Bu)_10_(FcCO_2_)_8_(MeCN)_4_]^0^·MeCN·2H_2_O	**CO**_**3**_**@Ag20f**	[CO_3_]^2–^	0	Stirring	2018	[206]
105	[CO_3_@Ag_20_(S^*t*^Bu)_10_(tfa)_8_(MeCN)_4_]^0^	**CO**_**3**_**@Ag20g**	[CO_3_]^2–^	0	Ultrasonication	2022	[204]
106	[CO_3_@Ag_20_(S^*t*^Bu)_10_(tfa)_8_(DMF)_3_(H_2_O)]^0^·DMF	**CO**_**3**_**@Ag20h**	[CO_3_]^2–^	0	Treating^*a*^	2022	[181]
107	[AsO_4_@Ag_20_(C≡C^*t*^Bu)_8_(Ph_2_PO_2_)_7_(tfa)_2_(MeCN)]^0^ ·4MeCN·2H_2_O	**AsO**_**4**_**@Ag20**	[AsO_4_]^3–^	0	Solvothermal	2020	[200]
108	[MoO_4_@Ag_20_(C≡C^*t*^Bu)_13_(tfa)_5_]^0^	**MoO**_**4**_**@Ag20**	[MoO_4_]^2–^	0	Stirring	2009	[186]
109	[[CO_3_@Ag_20_(S^*t*^Bu)_10_(OAc)_8_(DMF)_2_]·2H_2_O]_*n*_	**[CO**_**3**_**@Ag20a]**_***n***_	[CO_3_]^2–^	0	Stirring	2014	[197]
110	[CO_3_@Ag_20_(^*i*^PrS)_10_(NO_3_)_8_(DMF)_2_]_*n*_	**[CO**_**3**_**@Ag20b]**_***n***_	[CO_3_]^2–^	0	Solvothermal	2016	[196]
111	[[SO_4_@Ag_20_(S^*i*^Bu)_10_(PhSO_3_)_8_ (H_2_O)_4_]^0^·2H_2_O]_*n*_	**[SO**_**4**_**@Ag20a]**_***n***_	[SO_4_]^2–^	0	Solvothermal	2018	[137]
112	[SO_4_@Ag_20_(S^*i*^Pr)_10_(PTA)_3_(HPTA)_2_]_*n*_	**[SO**_**4**_**@Ag20b]**_***n***_	[SO_4_]^2–^	0	Solvothermal	2022	[202]
113	[SO_4_@Ag_21_(C≡C^*t*^Bu)_18_][BF_4_]	**SO**_**4**_**@Ag21**	[SO_4_]^2–^	+1	Stirring	2009	[187]
114	[CO_2_@Ag_20_Cu_2_S_2_(S^*t*^Bu)_10_(tfa)_8_(DMAc)_4_]^0^·DMAc	**CO**_**2**_**@Ag20Cu2**	[CO_2_]^0^	0	Stirring	2022	[181]
115	[CO_3_@Ag_22_(S^*t*^Bu)_10_(L-Ala)_4_(NO_3_)_6_(MeCN)_2_]_2_·H_2_O	**CO**_**3**_**@Ag22a**	[CO_3_]^2–^	0	Stirring	2018	[206]
116	[CO_3_@Ag_22_(S^*t*^Bu)_10_(D-Ala)_4_(NO_3_)_6_(MeCN)_2_]_2_·H_2_O	**CO**_**3**_**@Ag22b**	[CO_3_]^2–^	0	Stirring	2018	[206]
117	[NO_3_@Ag_22_(C≡C^*t*^Bu)_14_(^*t*^BuPO_3_)_2_(hfac)(tfa)(OH)]^0^ ·MeCN	**NO**_**3**_**@Ag22**	[NO_3_]^–^	0	Stirring	2018	[159]
118	[CrO_4_@Ag_22_(C≡C^*t*^Bu)_18_][BF_4_]_2_	**CrO**_**4**_**@Ag22**	[CrO_4_]^2–^	+2	Stirring	2009	[187]
119	[AsO_4_@Ag_22_(Ph_2_PO_2_)_7_(C≡C^*t*^Bu)_10_(MeOH)(H_2_O)_2_] [PF_6_]_2_	**AsO**_**4**_**@Ag22**	[AsO_4_]^3–^	0	Solvothermal	2020	[200]
120	[[SO_4_@Ag_22_(S^*i*^Pr)_12_(NO_3_)_6_][NO_3_]_2_]_*n*_	**[SO**_**4**_**@Ag22]**_***n***_	[SO_4_]^2–^	+2	Solvothermal	2016	[196]
121	[[CrO_4_@Ag_22_(C≡CPh)_16_(OTf)_4_]^0^]_*n*_	**[CrO**_**4**_**@Ag22]**_***n***_	[CrO_4_]^2–^	0	Solvothermal	2017	[136]
122	[CO_3_@Ag_23_(S^*t*^Bu)_10_(L-Val)_6_(NO_3_)_4_(MeCN)][OH]	**CO**_**3**_**Ag23a**	[CO_3_]^2–^	+1	Stirring	2018	[206]
123	[CO_3_@Ag_23_(S^*t*^Bu)_10_(D-Val)_6_(NO_3_)_4_(MeCN)][OH]	**CO**_**3**_**Ag23b**	[CO_3_]^2–^	+1	Stirring	2018	[206]
124	[CO_3_@Ag_24_(S^*t*^Bu)_10_(L-Pro)_8_(NO_3_)_4_(H_2_O)]^0^ ·4MeCN·3H_2_O	**CO**_**3**_**Ag24a**	[CO_3_]^2–^	0	Stirring	2018	[206]
125	[CO_3_@Ag_24_(S^*t*^Bu)_10_(D-Pro)_8_(NO_3_)_4_(H_2_O)]^0^ ·4MeCN·3H_2_O	**CO**_**3**_**Ag24b**	[CO_3_]^2–^	0	Stirring	2018	[206]
126	[(C_4_O_4_)_2_@Ag_24_(tfa)_4_(C≡C^*t*^Bu)_16_]·2MeCN	**C**_**4**_**O**_**4**_**Ag24**	[C_4_O_4_]^2–^	0	Solvothermal	2015	[205]
127	[MoO_4_@Ag_24_(SPhMe)_12_(dppm)_6_ (MoO_4_)_4_]^0^·2BF_4_·C_2_H_5_NO	**MoO**_**4**_**@Ag24a**	[MoO_4_]^2–^	0	Solvothermal	2017	[135]
128	[MoO_4_@Ag_24_(SPhMe)_12_(dppm)_6_ (MoO_4_)_4_]^0^·2OTf	**MoO**_**4**_**@Ag24b**	[MoO_4_]^2–^	0	Solvothermal	2017	[135]
129	[MoO_4_@Ag_24_(SPhMe)_12_(dppf)_6_(MoO_4_)_4_]^0^ ·2OTf	**MoO**_**4**_**@Ag24c**	[MoO_4_]^2–^	0	Solvothermal	2017	[135]
130	[MoO_4_@Ag_24_(SPhMe))_12_(dppb)_6_ (MoO_4_)_4_]^0^·2OTf	**MoO**_**4**_**@Ag24d**	[MoO_4_]^2–^	0	Solvothermal	2017	[135]
131	[(NO_3_)_2_@Ag_26_(*o*-MePhC≡C)_16_][NO_3_]_8_·5H_2_O	**(NO**_**3**_**)**_**2**_**@Ag26**	[NO_3_]^–^	+8	Stirring	2014	[185]
132	[VO_4_@Ag_26_(C≡C^*t*^Bu)_13_(tfa)_10_(MeCN)_2_ (MeOH)_3_]^0^·MeOH	**VO**_**4**_**@Ag26**	[VO_4_]^3–^	0	Solvothermal	2019	[142]
133	[(CO_3_)_2_@Ag_26_(C≡C^*t*^Bu)_16_{S_2_P(OEt)_2_}_4_][OH]_2_·4MeOH·6H_2_O	**(CO**_**3**_**)**_**2**_**@Ag26**	[CO_3_]^2–^	+2	Stirring	2018	[177]
134	[(CrO_4_)_2_@Ag_30_(SCH_2_Nap)_18_(DMAc)_2_(tfa)_8_]	**(CrO**_**4**_**)**_**2**_**@Ag30**	[CrO_4_]^2–^	0	Stirring	2019	[195]
135	[[(CrO_4_)_2_@Ag_31_(C≡CPh)_22_(OTf)_4_] [OTf]·2MeOH·H_2_O]_*n*_	**[CrO**_**4**_**@Ag31]**_***n***_	[CrO_4_]^2–^	+1	Solvothermal	2017	[136]
136	[(C_5_O_5_)_2_@Ag_32_S_2_{S_2_P(OEt)_2_}_22_][PF_6_]_2_	**(C**_**5**_**O**_**5**_**)**_**2**_**@Ag32**	[C_5_O_5_]^2–^	+2	Stirring	2022	[201]
137	[NO_3_@Ag_33_S_3_(S^*t*^Bu)_16_(tfa)_9_(MeCN)_2_][NO_3_]	**NO**_**3**_**@Ag33**	[NO_3_]^–^	+1	Stirring	2014	[190]
138	[(VO_4_)_2_@Ag_34_(C≡C^*t*^Bu)_22_(NO_3_)_6_]·8H_2_O	**(VO**_**4**_**)**_**2**_**@Ag34**	[VO_4_]^3–^	0	Stirring	2012	[198]
139	[(CrO_4_)_2_@Ag_35_(C≡CPh)_28_ (TMEDA)_4_][BF_4_]_3_	**(CrO**_**4**_**)**_**2**_**@Ag35**	[CrO_4_]^2–^	+3	Stirring	2009	[187]
140	[TeO_6_@Ag_36_(C≡C^*t*^Bu)_18_(tfa)_12_]	**TeO**_**6**_**@Ag36**	[TeO_6_]^6–^	0	Solvothermal	2020	[193]
141	[SO_4_@Ag_40_S_4_{S_2_P(OEt)_2_}_24_][PF_6_]_6_	**SO**_**4**_**@Ag40a**	[SO_4_]^2–^	+6	Stirring	2020	[194]
142	[SO_4_@Ag_40_Se_4_{S_2_P(OEt)_2_}_24_][PF_6_]_6_	**SO**_**4**_**@Ag40b**	[SO_4_]^2–^	+6	Stirring	2020	[194]
143	[[CO_3_@Ag_42_(C≡C^*t*^Bu)_27_(MeCN)_2_] [CoW_12_O_40_]_2_][BF_4_]	**CO**_**3**_**@Ag42a**	[CO_3_]^2–^	+1	Crystallization^*a*^	2010	[188]
144	[CO_3_@Ag_42_(C≡C^*t*^Bu)_27_(α-A-SiW_9_Nb_3_O_40_)_2_][Bu_4_N]	**CO**_**3**_**@Ag42b**	[CO_3_]^2–^	–1	Treating^*a*^	2015	[199]
145	[MoO_4_@Ag_12_@(^*n*^BuPO_3_)_8_S_6_@Ag_36_(S^*t*^Bu)_24_] [MeOH_2_]_6_	**MoO**_**4**_**@Ag48**	[MoO_4_]^2–^	–6	Stirring	2015	[172]
146	[(WO_4_)_2_(Ag_16_S_6_)@Ag_56_(C≡C^*t*^Bu)_27_(^*t*^BuPO_3_)_12_(OAc)(MeCN)_2_(H_2_O)]·[H_2_P_2_W_18_O_62_]^0^	**WO**_**4**_**@Ag72**	[WO_4_]^2–^	0	Solvothermal	2018	[158]
147	[SO_4_@Ag_78_S_15_(SC_5_H_9_)_27_(tfa)_12_][tfa]_7_	**SO**_**4**_**@Ag78**	[SO_4_]^2–^	−7	Crystallization^*a*^	2018	[189]

^*a*^See citation for detailed synthesis method.

**Table 5.** Representative POM-templated Ag NCs.

No.	Chemical formula	Abbreviation	Center anion	*z*	Synthesis method	Year	Ref.
148	[V_2_O_7_@Ag_22_(S^*t*^Bu)_8_{S_2_P(OEt)_2_}_9_][OH]·H_2_O	**POV@Ag22**	[V_2_O_7_]^4–^	+1	Stirring	2022	[204]
149	[V_2_O_7_@Ag_24_(C≡C^*t*^Bu)_14_(tfa)_6_]^0^·2MeOH	**POV@Ag24a**	[V_2_O_7_]^4–^	0	Stirring	2009	[186]
150	[V_2_O_7_@Ag_24_(C≡C^*t*^Bu)_14_(hfac)_6_]^0^	**POV@Ag24b**	[V_2_O_7_]^4–^	0	Ultrasonication	2018	[159]
151	[W_2_O_9_@Ag_24_(S^*t*^Bu)_14_{S_2_P(O^*i*^Pr)_2_}_6_]^0^·MeOH	**POW@Ag24**	[W_2_O_9_]^4–^	0	Stirring	2022	[204]
152	[P_2_W_15_Nb_3_O_62_@Ag_25_(C≡C^*t*^Bu)_16_(MeCN)_4_]^0^·11MeCN	**POW@Ag25**	[P_2_W_15_Nb_3_O_62_]^9–^	0	Crystallization^*a*^	2015	[222]
153	[V^V^_10_V^IV^_2_O_34_@Ag_30_(S^*t*^Bu)_20_]^0^·10MeOH	**POV@Ag30a**	[V^V^_10_V^IV^_2_O_34_]^10–^	0	Solvothermal	2016	[225]
154	[V^V^_10_V^IV^_2_O_34_@Ag_30_(S^*t*^Bu)_20_]^0^·7MeOH	**POV@Ag30b**	[V^V^_10_V^IV^_2_O_34_]^10–^	0	Stirring	2016	[225]
155	[V^V^_10_V^IV^_2_O_34_@Ag_30_(S^*t*^Bu)_20_]^0^·8MeOH	**POV@Ag30c**	[V^V^_10_V^IV^_2_O_34_]^10–^	0	Stirring	2016	[225]
156	[Mo_2_O_8_@Ag_30_(S^*i*^Bu)_15_(PhSO_3_)_11_(MeOH)_2_(H_2_O)·H_2_O]_2_	**[POMo** **@Ag30]**_**2**_	[Mo_2_O_8_]^4–^	0	Solvothermal	2018	[137]
157	[(W_6_O_21_)@Ag_34_(S^*t*^Bu)_26_(tfa)] [tfa]·Et_3_N·20MeOH	**POW@Ag34**	[W_6_O_21_]^6–^	+1	Stirring	2012	[209]
158	[SiW_9_O_34_@Ag_34_Cu_6_(C≡C^*t*^Bu)_18_(^*t*^BuPO_3_)_9_(MeCN)_2_(H_2_O)]^0^·2MeCN	**POW** **@Ag34Cu6**	[SiW_9_O_34_]^10–^	0	Solvothermal	2018	[158]
159	[((O_2_)V_2_O_6_)_2_@Ag_36_(C≡C^*t*^Bu)_12_{(^*t*^BuPO_3_)_3_V_4_O_8_}_2_ (^*t*^BuPO_3_)_2_(NO_3_)_7_(2-ClPy)(DMF)][Et_4_N]_3_	**POV@Ag36**	[(O_2_)V_2_O_6_]^4–^	–3	Stirring	2012	[152]
160	[(*α*-Mo_5_O_18_)@Ag_36_(S^*i*^Pr)_18_(PhSO_3_)_12_(DMF)_6_]^0^	**POMo@Ag36a**	[*α*-Mo_5_O_18_]^6–^	0	Solvothermal	2018	[137]
161	[(Mo_5_O_18_)@Ag_36_(S^*i*^Pr)_18_(O_3_SPhMe)_13.5_(MeCN)· 1.5MeCN][^*n*^Bu_4_N]_1.5_	**POMo@Ag36b**	[Mo_5_O_18_]^6–^	−1.5	Solvothermal	2018	[220]
162	[[^*n*^Bu_4_NH][(*β*-Mo_5_O_18_)@Ag_36_(S^*i*^Bu)_18_(PhSO_3_)_13_(MeOH)]^0^]_*n*_	**[POMo** **@Ag36]**_***n***_	[*β*-Mo_5_O_18_]^6–^	0	Solvothermal	2018	[137]
163	[SiW_9_O_34_@Ag_37_Cu_6_(C≡C^*t*^Bu)_18_(^*t*^BuPO_3_)_9_(MeCN)_6_] [SiW_12_O_40_]_0.5_[OH]	**POW** **@Ag37Cu6**	[SiW_9_O_34_]^10–^	+3	Solvothermal	2018	[158]
164	[*α*-Mo_5_O_18_@Ag_38_(^*t*^BuS)_18_(PhCO_2_)_14_·2(DCM)]^0^	**POMo@Ag38**	[*α*-Mo_5_O_18_]^6–^	0	Ultrasonication	2018	[219]
165	[V_10_O_28_@Ag_40_(C≡C^*t*^Bu)_22_(tfa)_12_]^0^·4MeOH	**POV@Ag40a**	[V_10_O_28_]^6–^	0	Stirring	2009	[186]
166	[V_2_O_7_@Ag_40_(C≡C^*t*^Bu)_22_(^*t*^BuPO_3_)_4_(hfac)_4_(tfa)_2_(H_2_O)_2_]^0^	**POV@Ag40b**	[V_2_O_7_]^4–^	0	Ultrasonication	2018	[159]
167	[Mo_6_O_22_@Ag_40_(C≡C^*t*^Bu)_20_(tfa)_12_]^0^·2MeOH	**POMo@Ag40**	[Mo_6_O_22_]^8–^	0	Stirring	2009	[186]
168	[*α*-SiW_10_O_37_@Ag_41_(C≡C^*t*^Bu)_27_(MeCN)_3_] [*β*-SiW_12_O_40_]·H_2_O·4MeCN	**POW@Ag41**	[*α*-SiW_10_O_37_]^10–^	–4	Solvothermal	2016	[216]
169	[(Mo_7_O_24_)@Ag_41_(S^*i*^Pr)_19_(*p*-tos)_16_(MeOH)_4_·4MeOH]^0^	**POMo@Ag41**	[Mo_7_O_24_]^6–^	0	Solvothermal	2018	[220]
170	[Ho(W_5_O_18_)_2_@Ag_42_(C≡C^*t*^Bu)_28_Cl_4_][OH]	**POW@Ag42a**	[Ho(W_5_O_18_)_2_]^9–^	+1	Solvothermal	2013	[210]
171	[EuW_10_O_36_@Ag_42_(C≡C^*t*^Bu)_28_(NO_3_)_4_·NO_3_]	**POW@Ag42b**	[EuW_10_O_36_]^9–^	0	Solvothermal	2018	[217]
172	[Eu(W_5_O_18_)_2_@Ag_42_(C≡C^*t*^Bu)_28_Cl_4_][OH]·H_2_O	**POW@Ag42**	[Eu(W_5_O_18_)_2_]^9–^	+1	Solvothermal	2015	[221]
173	[((O_2_)V_2_O_6_)_3_Ag_43_(C≡CPh)_19_{(^*t*^BuPO_3_)_4_V_4_O_8_}_3_ (DMF)_6_]^0^·5DMF·2H_2_O	**POV@Ag43**	[(O_2_)V_2_O_6_]^4–^	0	Stirring	2011	[184]
174	[K(H_2_O)HP_5_W_30_O_110_@Ag_43_(C≡C^*t*^Bu)_29_(CN)(MeCN)(H_2_O)]^0^·4MeCN	**POW@Ag43**	[K(H_2_O)HP_5_W_30_O_110_]^13–^	0	Solvothermal	2021	[227]
175	[(V_2_O_7_)_2_@Ag_44_(C≡C^*t*^Bu)_14_@(V_32_O_96_)][BzEt_3_N]_8_[Et_4_N]_2_	**POV@Ag44**	[V_2_O_7_]^4–^	–10	Stirring	2012	[224]
176	{[V_10_O_28_@Ag_44_(SEt)_20_(PhSO_3_)_18_(H_2_O)_2_]^0^}_*n*_	**[POV@Ag44]**_***n***_	[V_10_O_28_]^6–^	0	Solvothermal	2019	[138]
177	[Mo_6_O_22_@Ag_44_(S^*i*^Pr)_20_(PhCO_2_)_16_(MeCN)_2_]^0^ ·2MeCN	**POMo@Ag44**	[Mo_6_O_22_]^8–^	0	Solvothermal	2018	[139]
178	{[Mo_6_O_19_@Ag_44_(SEt)_24_(SCl_4_)_3_]^0^}_*n*_	**[POMo** **@Ag44]**_***n***_	[Mo_6_O_19_]^2–^	0	Solvothermal	2019	[138]
179	{[V_10_O_28_@Ag_46_(SEt)_23_(PhSO_3_)_15_(CO_3_)]^0^}_*n*_	**[POV@Ag46]**_***n***_	[V_10_O_28_]^6–^	0	Solvothermal	2019	[138]
180	[V_10_O_28_@Ag_46_(S^*i*^Pr)_28_(tfa)_12_(DMF)_2_]^0^	**POV@Ag46a**	[V_10_O_28_]^6–^	0	Solvothermal	2022	[202]
181	[V_10_O_28_@Ag_46_(S^*i*^Pr)_30_(tfa)_8_(PhCO_2_)_2_(DMF)_4_]^0^	**POV@Ag46b**	[V_10_O_28_]^6–^	0	Solvothermal	2022	[202]
182	[Mo_6_O_22_@Ag_46_(SPh^*t*^Bu)_32_(dppm)_4_(MeCN)_8_][OTf]_6_	**POMo@Ag46**	[Mo_6_O_22_]^8–^	+6	Solvothermal	2017	[135]
183	[Mo_8_O_28_@Ag_48_(*p*-MePhS)_24_ (tfa)_14_(H_2_O)_4_(DMF)_2_][tfa]_2_	**POMo@Ag48**	[Mo_8_O_28_]^8–^	+2	Solvothermal	2019	[140]
184	[V_10_O_28_@Ag_50_(SPh^*t*^Bu)_32_(tfa)_8_(DMF)_6_(H_2_O)_4_][tfa]_4_ ·12DMF	**POV@Ag50**	[V_10_O_28_]^6–^	+4	Solvothermal	2022	[228]
185	[Mo_8_O_28_@Ag_50_(S^*i*^Pr)_24_(PhCO_2_)_18_(MeCN)_2_]]^0^ ·4MeCN	**POMo@Ag50a**	[Mo_8_O_28_]^8–^	0	Solvothermal	2018	[139]
186	[Mo_8_O_28_@Ag_50_(S^*i*^Pr)_24_(4-MePhCO_2_)_14_(PhCO_2_)_4_(MeCN)_4_]^0^	**POMo@Ag50b**	[Mo_8_O_28_]^8–^	0	Solvothermal	2018	[139]
187	[Mo_8_O_28_@Ag_50_(S^*i*^Pr)_24_(4-MePhCO_2_)_16.5_(PhCO_2_)_1.5_]^0^	**POMo@Ag50c**	[Mo_8_O_28_]^8–^	0	Solvothermal	2018	[139]
188	[Mo_8_O_28_@Ag_50_(S^*i*^Pr)_24_(3-MePhCO_2_)_18_ (3-MePhCO_2_H)(MeCN)_2_]^0^	**POMo@Ag50d**	[Mo_8_O_28_]^8–^	0	Solvothermal	2018	[139]
189	[(WO_6_)(SiW_9_O_34_)@Ag_51_(C≡C^*t*^Bu)_27_(^*n*^BuPO_3_)_3_(OAc)_3_(MeCN)_3_]·0.5[H_8_SiW_12_O_40_][BF_4_]	**POW@Ag51**	[(WO_6_)(SiW_9_O_34_)]^16–^	+1	Solvothermal	2018	[158]
190	[Cr^III^_4_Cr^VI^_8_O_36_@Ag_56_(S^*i*^Pr)_28_(tfa)_12_(NapCO_2_)_4_(MeCN)_8_]^0^·2MeCN	**POCr@Ag56**	[Cr^III^_4_Cr^VI^_8_O_36_]^12–^	0	Solvothermal	2020	[226]
191	[Mo_6_O_22_@Ag_58_S_2_(SPh^*t*^Bu)_36_(tfa)_10_(H_2_O)_8_]^0^	**POMo@Ag58**	[Mo_6_O_22_]^8–^	0	Solvothermal	2015	[214]
192	[(Mo_6_O_22_)_2_Ag_60_(C≡C^*t*^Bu)_38_][OTf]_6_	**POMo@Ag60**	[Mo_6_O_22_]^8–^	+6	Solvothermal	2010	[211]
193	[Mo_20_O_66_@Ag_62_(S^*t*^Bu)_40_(Mo_6_O_19_)_3_(MeCN)_2_][OTf]_4_	**POMo@Ag62**	[Mo_20_O_66_]^12–^	+4	Stirring	2015	[215]
194	[(Mn^III^Mn^IV^_2_Mo_14_O_56_)@Ag_64_(C≡C^*t*^Bu)_38_ (tfa)_8_][OH]·10MeCN·2H_2_O	**POMo@Ag64**	[Mn^III^Mn^IV^_2_Mo_14_O_56_]^17–^	+1	Solvothermal	2016	[212]
195	[(PW_9_O_34_)_2_@Ag_67_(*p*-FPhS)_36_(DMAc)_2_(tfa)_6_][tfa]_7_·(DMAc)_*x*_	**POW@Ag67**	[PW_9_O_34_]^9–^	+7	Stirring	2019	[223]
196	[(PW_9_O_34_)_2_@Ag_70_(C≡C^*t*^Bu)_44_(H_2_O)_2_][BF_4_]_8_ ·2[BMIm]BF_4_·3H_2_O	**POW@Ag70**	[PW_9_O_34_]^9–^	+8	Solvothermal	2014	[213]
197	[(EuW_10_O_36_)_2_@Ag_72_(C≡C^*t*^Bu)_48_Cl_2_·4BF_4_]	**POW@Ag72**	[EuW_10_O_36_]^9–^	0	Solvothermal	2018	[217]
198	[(Mo_6_O_22_)_2_@Ag_76_(SPhOMe)_28_(dppm)_8_ (MoO_4_)_16_(H_2_O)_8_·8MeOH·4MeCN]^0^	**POMo@Ag76**	[Mo_6_O_22_]^8–^	0	Ultrasonication	2018	[218]

^*a*^See citation for detailed synthesis method.

**Table 6.** Representative H/D-templated Ag NCs.

No.	Chemical formula	Abbreviation	Center anion	*z*	Synthesis method	Year	Ref.
199	(Δ)_4_-[Na]_9_[H@Ag_4_{Rh(L-Cys)_3_}_4_]·*n*H_2_O	**H@Ag4Rh4**	H^–^	–9	Stirring	2021	[240]
200	(Δ)_4_-[Na]_9_[D@Ag_4_{Rh(L-Cys)_3_}_4_]·*n*H_2_O	**D@Ag4Rh4**	H^–^	–9	Stirring	2021	[240]
201	[H@Ag_7_{Se_2_P(O^*i*^Pr)_2_}_6_]^0^	**H@Ag7a**	H^–^	0	Stirring	2013	[235]
202	[H@Ag_7_{S_2_P(OEt)_2_}_6_]^0^	**H@Ag7b**	H^–^	0	Stirring	2013	[235]
203	[H@Ag_7_{S_2_C≡C(CN)_2_}_6_][Bu_4_N]_6_	**H@Ag7c**	H^–^	–6	Stirring	2019	[238]
204	[D@Ag_7_{Se_2_P(O^*i*^Pr)_2_}_6_]^0^	**D@Ag7a**	H^–^	0	Stirring	2013	[235]
205	[D@Ag_7_{S_2_P(OEt)_2_}_6_]^0^	**D@Ag7b**	H^–^	0	Stirring	2013	[235]
206	[D@Ag_7_{S_2_C≡C(CN)_2_}_6_][Bu_4_N]_6_	**D@Ag7c**	D^–^	–6	Stirring	2019	[238]
207	[H@Ag_8_{Se_2_P(O^*i*^Pr)_2_}_6_][PF_6_]	**H@Ag8a**	H^–^	+1	Stirring	2010	[236]
208	[H@Ag_8_{Se_2_P(OEt)_2_}_6_][PF_6_]	**H@Ag8b**	H^–^	+1	Stirring	2010	[236]
209	[H@Ag_8_{S_2_P(OEt)_2_}_6_][PF_6_]	**H@Ag8c**	H^–^	+1	Stirring	2010	[132]
210	[H@Ag_8_{S_2_C≡C(CN)_2_}_6_][Bu_4_N]_5_	**H@Ag8d**	H^–^	–5	Stirring	2011	[239]
211	[H@Ag_8_{S_2_P(O^*i*^Pr)_2_}_6_][PF_6_]	**H@Ag8e**	H^–^	+1	Stirring	2014	[133]
212	[D@Ag_8_{Se_2_P(O^*i*^Pr)_2_}_6_][PF_6_]	**D@Ag8a**	D^–^	+1	Stirring	2010	[236]
213	[D@Ag_8_{Se_2_P(OEt)_2_}_6_][PF_6_]	**D@Ag8b**	D^–^	+1	Stirring	2010	[236]
214	[D@Ag_8_{S_2_P(OEt)_2_}_6_][PF_6_]	**D@Ag8c**	D^–^	+1	Stirring	2010	[132]
215	[D@Ag_8_{S_2_C≡C(CN)_2_}_6_][Bu_4_N]_5_	**D@Ag8d**	D^–^	–5	Stirring	2011	[239]
216	[H@Ag_11_(S_5_CNPr_2_)_9_][NO_3_]	**H@Ag11**	H^–^	+1	Stirring	2011	[237]
217	[D@Ag_11_(S_2_CNPr_2_)_9_][NO_3_]	**D@Ag11**	D^–^	+1	Stirring	2011	[237]

In Section 2, we describe the synthesis methods for anion-templated Ag NCs. In Section 3, we describe the results of the synthesis and geometric structures of the anion-templated Ag NCs while categorizing them by anion species. After a short conclusion in Section 4, the challenges and future avenues for the development of anion-templated Ag NCs are described in Section 5.

## 2. Synthesis methods

**Figure 2.**
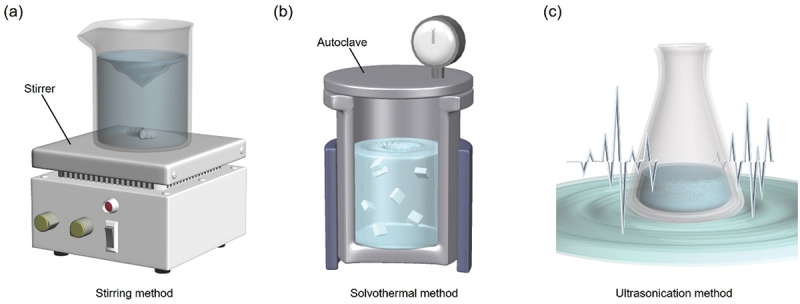
Three representative methods in the synthesis of anion-templated Ag NCs. (a) Stirring method, (b) solvothermal method, and (c) ultrasonication method.

Anion-templated Ag NCs are generally synthesized by mixing Ag salts and ligand compounds in solution and adding a reducing agent or other agents to the solution. The addition of a reducing agent is not essential, and in some cases, anions can be formed without a reducing agent [131]. SR, PR_3_, and C≡CR are frequently used as ligands because of their high affinity to Ag. During synthesis, a precursor, which induces the formation of a template anion, is generally added. However, the addition of precursors is also not essential, and in some cases, anions can be formed without adding precursors, as in the case of Cl@Cu_14_(SC(CH_3_)_2_CH_2_NH_2_)_12_ described in Section 1. In both cases, anion-templated Ag NCs cannot form unless the conditions are conducive to the generation of a central anion. In most cases, anions are formed by stirring the mixture to induce a chemical reaction (stirring method, Figure 2(a)). The solvothermal (Figure 2(b)) and ultrasonication methods (Figure 2(c)) are also often used.

The stirring method is the most frequently used, and the majority of anion-templated Ag NCs are synthesized by this method. In this method, specific central anions are produced by simple stirring and the addition of reaction accelerators. Stirring is generally performed at temperatures from −20 to 25 °C [131–134]. When CO_3_^2–^ is used as a template source, atmospheric CO_2_ can also be used, where CO_2_ is converted to CO_3_^2–^ by adding *N,N,N’,N’*-tetramethylethylenediamine (TMEDA, Table 1) to the reaction system: in this reaction, the basicity of the solvent is important to produce CO_3_^2–^ from H_2_CO_3_ formed by hydration of CO_2_ [125]. Solvothermal methods are also frequently used in the synthesis of anion-templated Ag NCs [135–140]. In these methods, the chemical species in the reaction system are thermally decomposed to produce central anions. Typically, the heating is conducted at lower temperatures (60–80 °C) than those used for the synthesis of metal nanoparticles. The reason is that high temperatures might result in the decomposition of Ox and POM.

In the ultrasonication method, a chemical source of the template anion is dissolved in a solvent, which is then sonicated to induce the formation of anions. Using the ultrasonication method, the reaction solution can be heated and cooled more rapidly compared with the solvothermal method, allowing different reactions to proceed [141–144].

## 3. Examples of anion-templated Ag NCs

In the following sections, we describe the syntheses and geometric structures of anion-templated Ag NCs containing X (X@Ag_*n*_ NCs, Section 3.1, Figure 1(a)), X’ (X’@Ag_*n*_ NCs, Section 3.2, Figure 1(b)), Ox (Ox@Ag_*n*_ NCs, Section 3.3, Figure 1(c)), POM (POM@Ag_*n*_ NCs, Section 3.4, Figure 1(d)), and H/D (H/D@Ag_*n*_ NCs, Section 3.5, Figure 1(e)). The abbreviations for the ligands, functional groups, and solvents used in this review are summarized in Table 1.

### 3.1. Halide ion-templated Ag NCs

Table 2 summarizes the reported X@Ag_*n*_ NCs and includes their abbreviations [131–133,145–163]. In the following section, we describe their syntheses and the effects of the different X species on the geometric structures of Ag NCs.

#### 3.1.1. X@Ag_8_NCs

There are numerous reports on X@Ag_8_ NCs with a diverse range of central anions, including fluoride ions (F^–^), Cl^–^, and bromide ions (Br^–^). All these X@Ag_8_ NCs have been synthesized by Liu and colleagues. They reported the syntheses of **2** (**F@Ag8a**), **3** (**F@Ag8b**), **4** (**Cl@Ag8a**), **5** (**Cl@Ag8b**), **6** (**Cl@Ag8c**), **7** (**Cl@Ag8d**), **8** (**Cl@Ag8e**), **9** (**Br@Ag8a**), **10** (**Br@Ag8b**), **11** (**Br@Ag8c**), and **12** (**Br@Ag8d**) from 2004 to 2014 [131–133]. All of these X@Ag_8_ NCs were synthesized using the stirring method. For the precursors of each X, tetrabutylammonium fluoride (Bu_4_NF), tetrabutylammonium chloride (Bu_4_NCl), tetrabutylammonium bromide (Bu_4_NBr), benzyltriethylammonium chloride ((PhCH_2_)Et_3_NCl), or tetraphenylphosphonium bromide (PPh_4_Br) were used, and the central X anion is formed by their dissociation in the reaction process.

**Figure 3.**
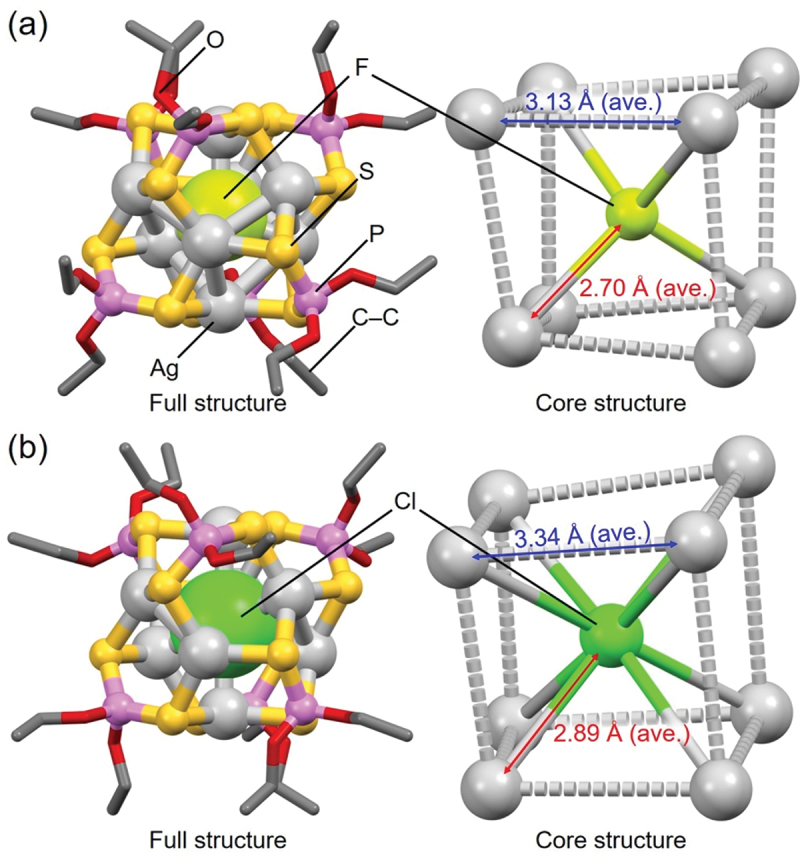
Full (left) and core (right) structures of (a) 2 (**F@Ag8a**) and (b) 7 (**Cl@Ag8d**). Data are taken from ref [132].

These eleven X@Ag_8_ NCs all contain the same number of Ag atoms, and their geometric structures are similar; they all have a distorted cubic framework composed of eight Ag atoms, with X located at the central position (Figure 3). However, the X–Ag distance differs slightly depending on the central X anion. For example, in **2** (**F@Ag8a**, Figure 3(a)), the average X–Ag distance from the central X anion to the surrounding core Ag is 2.70 Å, whereas the distance is 2.89 Å in **7** (**Cl@Ag8d**, Figure 3(b)). There is also a difference in the average Ag–Ag distance within the Ag_8_ core between **2** and **7**; it is 3.13 Å for **2** (**F@Ag8a**, Figure 3(a)) and 3.34 Å for **7** (**Cl@Ag8d**, Figure 3(b)). These results indicate that a smaller central X anion results in a more contracted X@Ag_8_ NCs framework. Furthermore, only four Ag atoms form bonds with the central F^–^ in **2** (**F@Ag8a**), whereas eight Ag atoms bond to the central Cl^–^ in **7** (**Cl@Ag8d**). Thus, a different type of central X anion induces a slight difference in the size of the X@Ag_8_ NC framework and the number of bonds within the framework.

**Figure 4.**
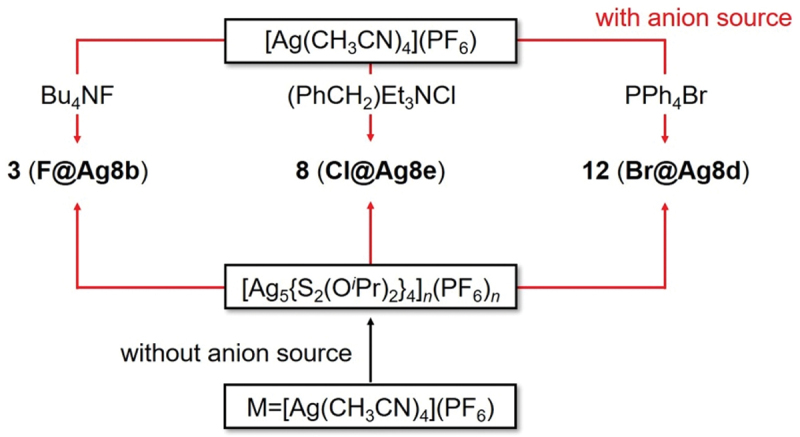
Two synthesis methods for 3 (**F@Ag8b**), 8 (**Cl@Ag8e**), and 12 (**Br@Ag8d**). Red lines are methods using anion sources. Black ones are methods without any anion sources. Data are taken from ref [133]. Reprinted with permission from [133], copyright (2014, the Royal Society of Chemistry).

Moreover, in the synthesis performed by Liu and colleagues, under conditions without an anion source, one-dimensional structures of [Ag_5_(S_2_P(O^*i*^Pr)_2_)_4_]_*n*_(PF_6_)_*n*_ were formed instead of X@Ag_8_ NCs. However, it has been shown that the addition of an anion source to the resulting [Ag_5_(S_2_P(O^*i*^Pr)_2_)_4_]_*n*_(PF_6_)_*n*_ leads to the formation of **3** (**F@Ag8b**), **8** (**Cl@Ag8e**), and **12** (**Br@Ag8d**) (Figure 4).

#### 3.1.2. X@Ag_14_NCs

The synthesis of X@Ag_14_ NC was reported by Rais and colleagues in 2001 [147] and 2002 [148]. These X@Ag_14_ NCs are pioneering examples of Ag NCs containing X. In Table 2, **20** (**F@Ag14**), **21** (**Cl@Ag14a**), **22** (**Cl@Ag14b**), and **23** (**Br@Ag14**) correspond to these X@Ag_14_ NCs, most of which were synthesized by the stirring method. In the synthesis of **21** (**Cl@Ag14a**), Ag-containing polymer compound [Ag(C≡C^*t*^Bu)]_*n*_ was used as an Ag precursor, whereas silver tetrafluoroborate (AgBF_4_) was used as an Ag precursor in the synthesis of **20** (**F@Ag14**), **22** (**Cl@Ag14b**), and **23** (**Br@Ag14**). The F^–^ in **20** (**F@Ag14**) was generated by the hydrolysis reaction [164,165] of the Ag precursor (AgBF_4_). For **21** (**Cl@Ag14a**) and **22** (**Cl@Ag14b**), the Cl^–^ was generated by the dissociation of the chloroform (the reaction solvent) and Bu_4_NCl added as the Cl^–^ source, respectively. In the synthesis of **23** (**Br@Ag14**), Bu_4_NBr was added as the Br^–^ source.

**Figure 5.**
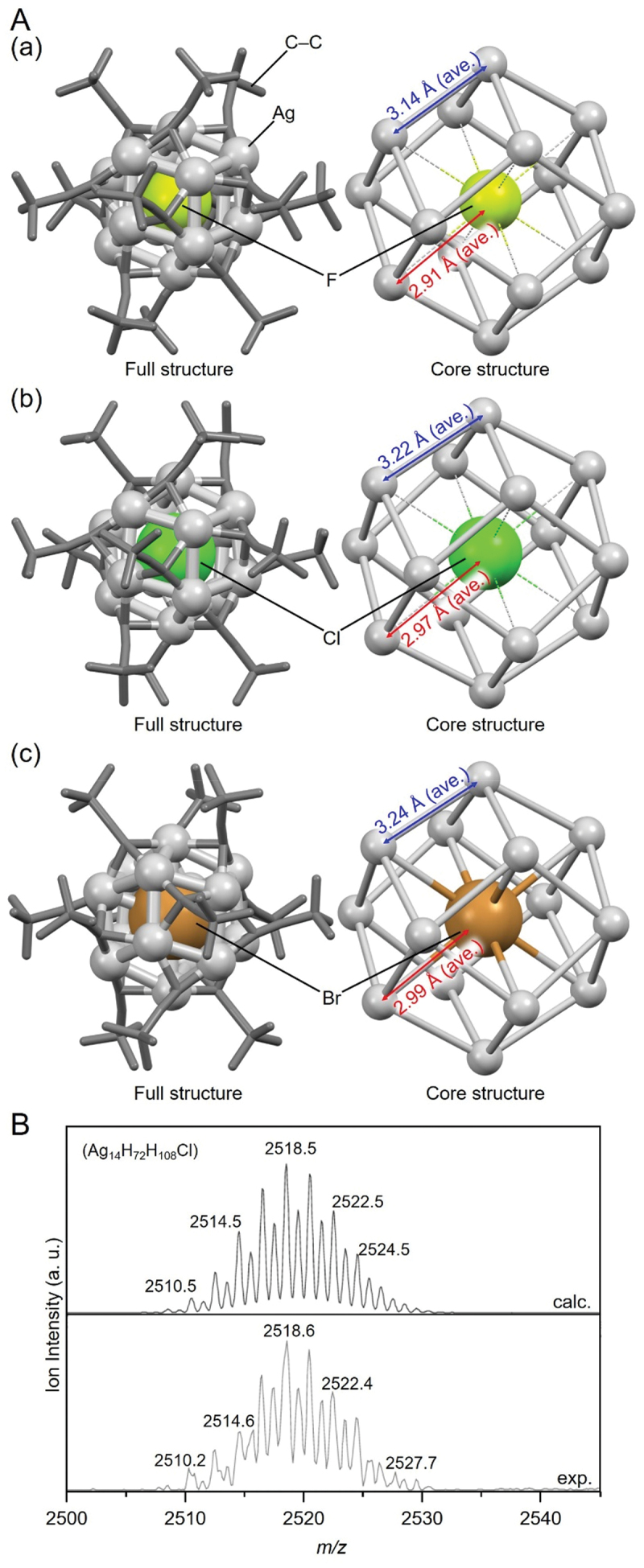
(A) Full (left) and core (right) structures of (a) 20 (**F@Ag14**), (b) 21 (**Cl@Ag14a**), and (c) 23 (**Br@Ag14**). (B) the observed and simulated ESI-MS pattern for 21 (**Cl@Ag14a**). Data are taken from refs [147,148]. Reprinted with permission from [147], copyright (2001, WILEY-VCH Verlag GmbH, Weinheim, Fed. Rep. of Germany).

Figure 5A shows the geometric structures of **20** (**F@Ag14**, Figure 5A(a)), **21** (**Cl@Ag14a**, Figure 5A(b)), and **23** (**Br@Ag14**, Figure 5A(c)), as determined by single-crystal X-ray diffraction (SC-XRD). All of the X@Ag_14_ NCs have a cubic framework consisting of eight Ag atoms, with an additional Ag atom on each of the six faces of the cube. This results in the construction of a rhombic dodecahedral Ag framework. Similar to X@Ag_8_ NCs, the size of the Ag framework in X@Ag_14_ NCs varies depending on the X at the central position. For example, for **20** (**F@Ag14**), **21** (**Cl@Ag14a**), and **23** (**Br@Ag14**), the average X–Ag distances are 2.91, 2.97, and 2.99 Å, respectively, and the average Ag–Ag distances are 3.14, 3.22, and 3.24 Å, respectively. The X–Ag distances in these X@ Ag_14_ NCs (X = F, Cl, and Br) are longer than those in the AgF (2.46 Å) [166], AgCl (2.77 Å) [167], and AgBr (2.89 Å) salts [167], indicating that the free volume exists in the Ag_14_ cage. The free volume decreases with the increasing ionic radius of X.

For **20** (**F@Ag14**), **22** (**Cl@Ag14b**), and **23** (**Br@Ag14**), electrospray ionization mass spectrometry (ESI-MS) has been performed, in addition to SC-XRD. Characteristic peaks appear in the spectra of **22** (**Cl@Ag14b**) and **23** (**Br@Ag14**) (Figure 5B), which means that these NCs can maintain their chemical composition in solution. In contrast, no peak has been observed at the position that can be attributed to **20** (**F@Ag14**), implying that **20** (**F@Ag14**) degrades in solution.

#### 3.1.3. X@Ag_16_NCs

**Figure 6.**
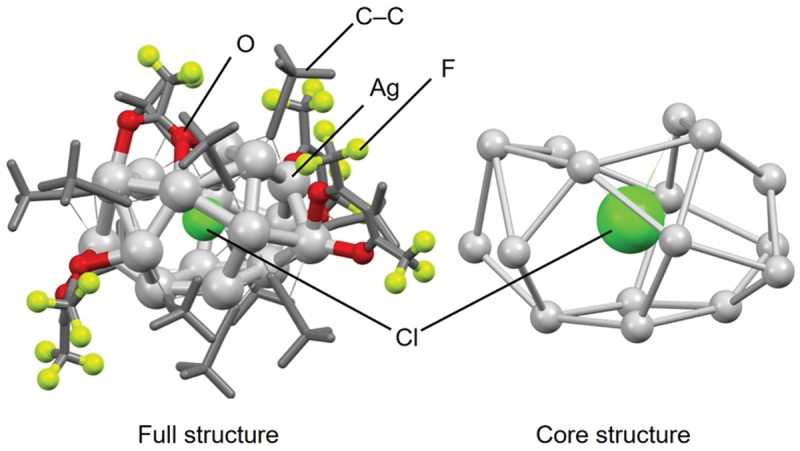
Full (left) and core (right) structures of 29 (**Cl@Ag16c**). Data are taken from ref [159].

The synthesis of X@Ag_16_ NC was also often reported. In Table 2, **27** (**Cl@Ag16a**), **28** (**Cl@Ag16b**), **29** (**Cl@Ag16c**), **30** (**Cl@Ag16d**), and **31** (**Cl@Ag16e**) correspond to these X@Ag_16_ NCs. Among them, **27**, **28**, **30**, and **31** were prepared by the stirring method, whereas **29** was prepared by the ultrasonication method. Figure 6 shows the geometrical structure of **29** (**Cl@Ag16c**). This NC encapsulates a template chloride anion, with argentophilic Ag⋯Ag bond distances in the range of 2.703–3.286 Å. The chloride ion coordinates to three silver atoms in this structure. The X@Ag_16_ core has ellipsoid structure. There structures are largely different from the above-described X@Ag_8_ and X@Ag_14_ NCs. Thus, the structure of X@Ag_*n*_ core strongly changes depending on the nymber of Ag atoms.

#### 3.1.4. Other X@Ag_n_NCs

At the end of this section, we introduce the syntheses of X@Ag_*n*_ NCs that are different from the Ag NCs described in Sections 3.1.1 and 3.1.2.

**Figure 7.**
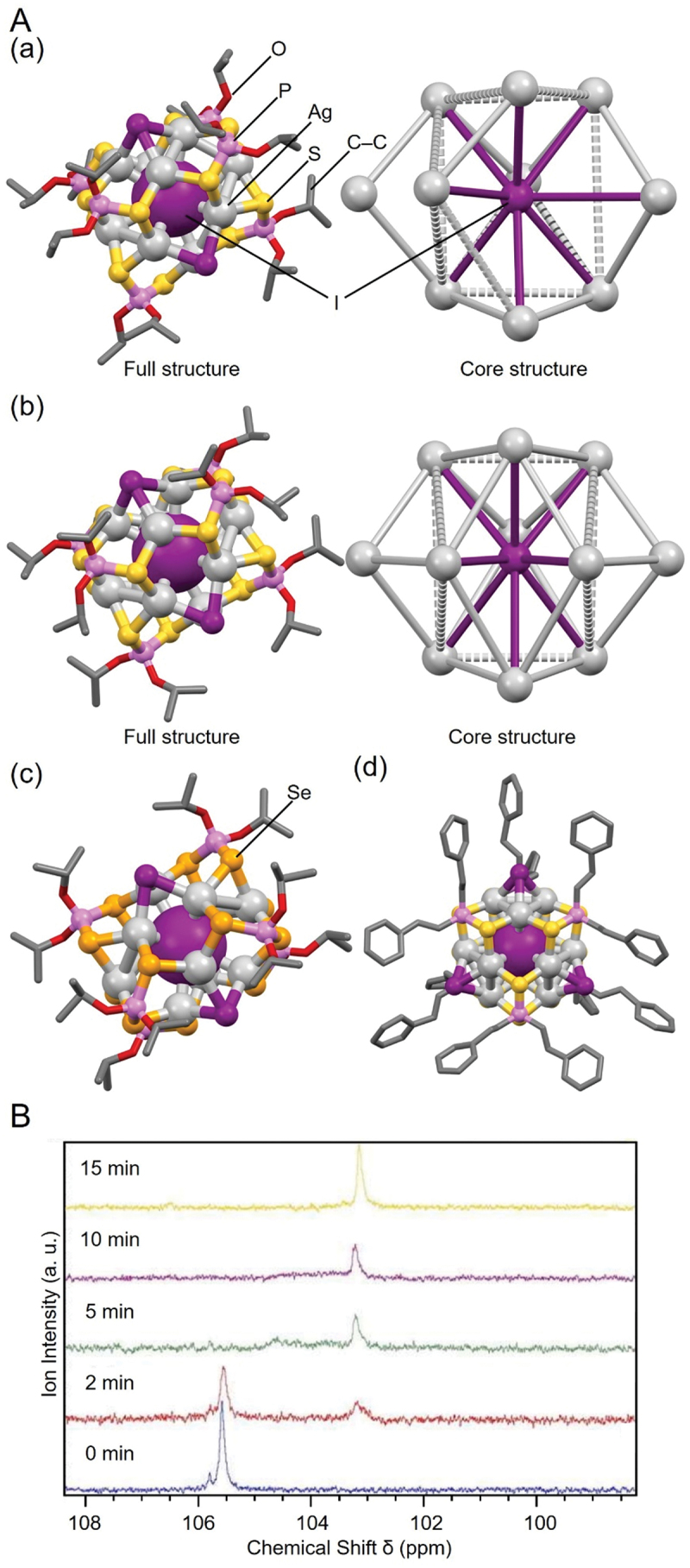
(A) Full (left) and core (right) structures of (a) 13 (**I@Ag10**), (b) 14 (**I@Ag11a**), (c) 15 (**I@Ag11b**), and (d) 17 (**I@Ag12**). (B) the ^31^P {^1^H} NMR spectra of monitoring the conversion within 15 minutes from 13 (**I@Ag10**) to 14 (**I@Ag11a**) by adding one equivalent of [Ag(CH_3_CN)_4_][PF_6_] relative to the case of the synthesis of 13 (**I@Ag10**). Data are taken from refs [133,145,157]. Reprinted with permission from [133], copyright (2014, the Royal Society of Chemistry).

The first example is the I@Ag_*n*_ NC, containing an iodide ion (I^–^). Representative examples of I@Ag_*n*_ NCs include **13** (**I@Ag10**) [133], **14** (**I@Ag11a**), **15** (**I@Ag11b**) [157], and **17** (**I@Ag12**) [145] (Figure 7A). All of these I@Ag_*n*_ NCs have been synthesized by Liu and colleagues. They used tetrabutylammonium iodide (Bu_4_NI) as the I^–^ precursor and the stirring method for synthesis. In the syntheses of **13** (**I@Ag10**) and **14** (**I@Ag11a**), although the same ligands were used, an excess of one equivalent of the Ag precursor was added in the synthesis of **14** (**I@Ag11a**) over the case of **13** (**I@Ag10**). They tracked the effect of this excess Ag precursor on the chemical composition of the product by multinuclear (^1^H and ^31^P) magnetic resonance spectroscopy (^31^P {^1^H} NMR). The results demonstrated that **14** (**I@Ag11a**) begins to form approximately 5 minutes after initiation of the reaction (Figure 7B). Comparing the geometric structures, **13** (**I@Ag10**) has a cage structure with one Ag atom missing from the Ag_11_ cage of **14** (**I@Ag11a**). The addition of an extra Ag precursor caused the addition of an Ag atom to the missing site, resulting in the conversion of **13** (**I@Ag10**) to **14** (**I@Ag11a**). From this observation, they suggested that NCs with one Au atom added to **13** (**I@Ag10**) could be obtained by adding the compound that would serve as an Au precursor to the solution of **13** (**I@Ag10**).

**Figure 8.**
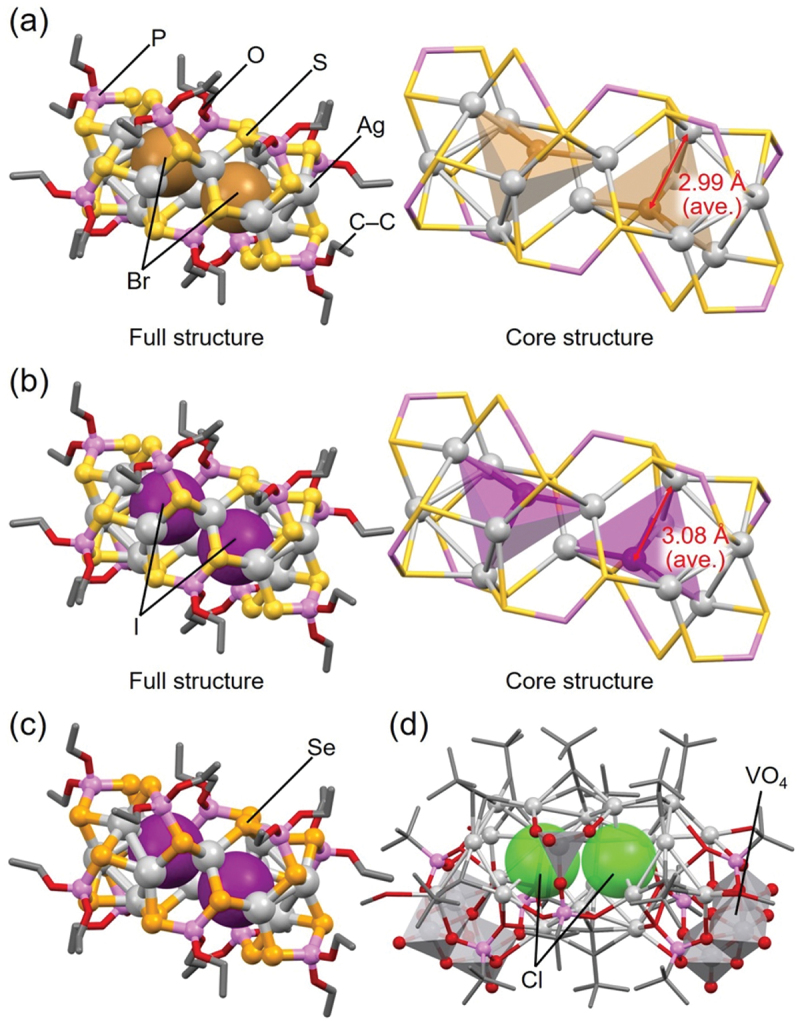
Full (left) and core (right) structures of (a) 16 (**Br_2_@Ag12**) and (b) 18 (**I_2_@Ag12a**). Full structures of (c) 19 (**I_2_@Ag12b**) and (d) 36 (**Cl2@Ag21**). Data are taken from refs [146,152].

The second example is the X_2_@Ag_*n*_ NC, in which two X anions are encapsulated within one Ag framework. Liu and colleagues reported the syntheses of **16** (**Br**_**2**_**@Ag12**), **18** (**I**_**2**_**@Ag12a**), and **19** (**I**_**2**_**@Ag12b**) [146] in 2013, and Xie and Mak reported the synthesis of **36** (**Cl**_**2**_**@Ag21**) [152] in 2012 (Table 2). These X_2_@Ag_*n*_ NCs are generally similar in shape, and all of them have peanut- or butterfly-shaped frameworks composed of Ag and S atoms (Figure 8). The average X–Ag distance in **16** (**Br**_**2**_**@Ag12**) is 2.80 Å, which is longer than the average X–Ag distance (2.98 Å) in **18** (**I**_**2**_**@Ag12a**), protected by the same ligand. This indicates that the X–Ag distance also varies with the size of the central X anion in the case of X_2_@Ag_*n*_ NCs, similar to X@Ag_8_ and X@Ag_14_ NCs.

### 3.2. Chalcogenide ion-templated Ag NCs

In this section, we describe the X’@Ag_*n*_ NCs that encapsulate X’ at the central position. Table 3 summarizes the representative X’@Ag_*n*_ NCs [124,133–135,137,143,144,168–182]. Again, to discuss the effects of the central X’ species on the geometric structure of the X’@Ag_*n*_ NCs, we limit the examples to the X’@Ag_9_ NCs and X’@Ag_14_ NCs.

#### 3.2.1. X’@Ag_9_ NCs

For X’@Ag_9_ NCs, the synthesis of **42** (**S@Ag9**) and **43** (**Se@Ag9**) with S^2–^ or selenide ion (Se^2–^) at the central position has been reported by Liu and colleagues in 2017 [134] (Table 3). Both X’@Ag_9_ NCs were synthesized by mixing the Ag precursor, tetrakis(acetonitrile)copper(I) hexafluorophosphate (Ag(MeCN)_4_PF_6_), and the ligand precursor, chalcogen-containing ammonium salt (NH_4_X’_2_P(OEt)_2_, X’ = S or Se), in acetone, followed by the addition of NaX’H (X’ = S or Se), which was then stirred at a low temperature (−20 or 0 °C). For the synthesis of **42** (**S@Ag9**), a polymer compound ([Ag_5_(S_2_P(OEt)_2_)_4_(PF_6_)]_*n*_) can be used as a precursor, instead of the ligand precursor.

**Figure 9.**
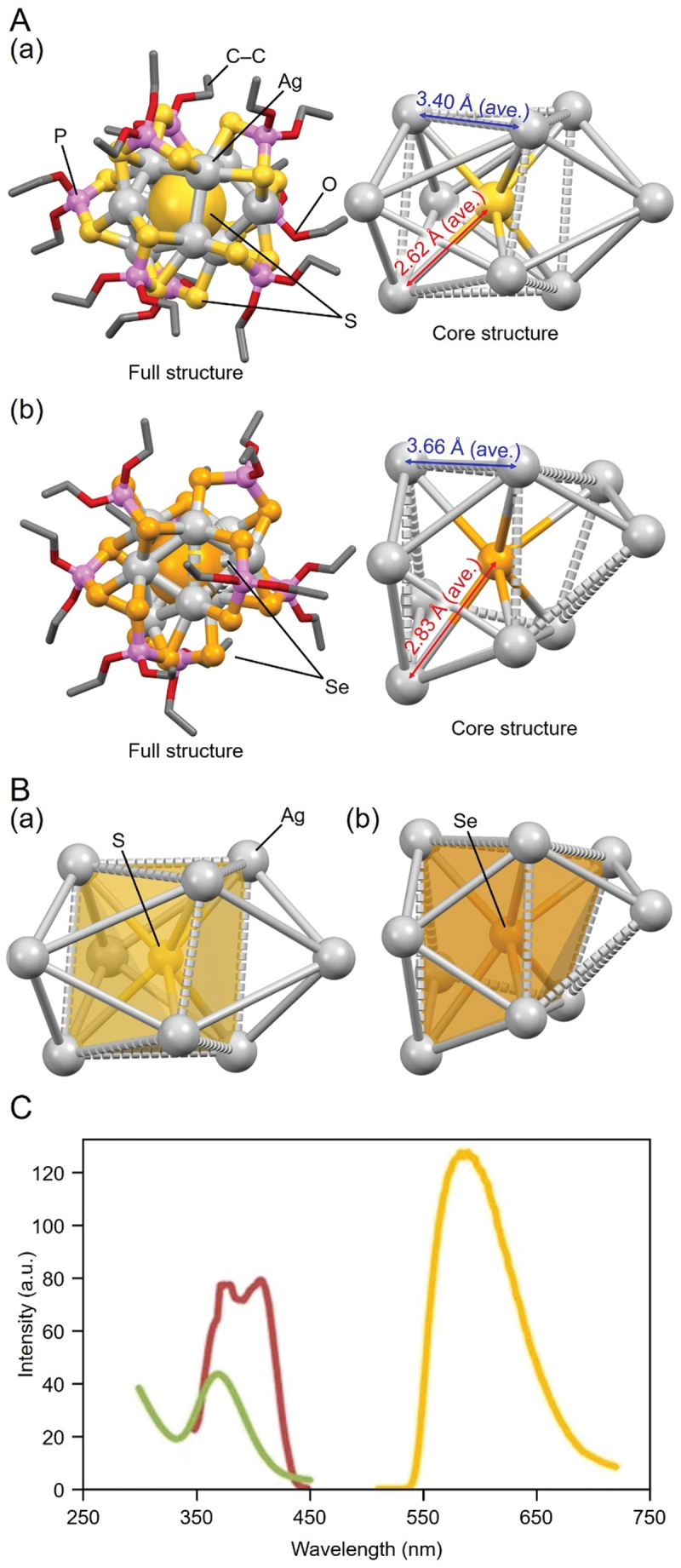
(A) Full (left) and core (right) structures of (a) 42 (**S@Ag9**) and (b) 43 (**Se@Ag9**). (B) Illustration on the differences in distortion in the a and B core structures. (C) Absorption (green, *ε* = 8760 (M^–1^ cm^–1^) and photoluminescence spectra (excitation, red; emission, orange) of 42 (**S@Ag9**) at 77 K. Data are taken from ref [134]. Reprinted with permission from [123], copyright (2016, Springer Science Business Media New York).

Figure 9A shows the geometric structures of **42** (**S@Ag9**) and **43** (**Se@Ag9**), as determined by SC-XRD [134] Both X’@Ag_9_ NCs have a geometric structure with three Ag atoms surrounding an hourglass-shaped Ag_6_ core. However, there are also differences between them. For example, focusing on the hourglass-shaped Ag_6_ core, the average Ag–Ag distance is slightly shorter in **42** (**S@Ag9**) than in **43** (**Se@Ag9**), with 3.40 and 3.66 Å, respectively. The average X’–Ag distance is also slightly shorter for **42** (**S@Ag9**) compared with **43** (**Se@Ag9**), exhibiting 2.62 and 2.83 Å, respectively. These factors lead to a slightly different distortion of the Ag_9_ framework (Figure 9B)

The different central X’ anions also affect the electronic structures of Ag NCs [134]. As shown in Figure 9C, a peak is observed at 370 nm in the optical absorption spectrum of **42** (**S@Ag9**), whereas peaks appear around 400 and 450 nm in the optical absorption spectrum of **43** (**Se@Ag9**) (Figure 9C) [134]. These differences are due to the varying degrees of charge transfer between the central X’ anion and the Ag framework.

#### 3.2.2. X’@Ag_14_ NCs

For X’@Ag_14_ NCs, **54** (**S@Ag14a**), **55** (**S@Ag14b**), **56** (**S@Ag14c**), **57** (**S@Ag14d**), and **60** (**Se@Ag14**) have been reported (Table 3). The former examples, **54** (**S@Ag14a**), **55** (**S@Ag14b**), and **56** (**S@Ag14c**) and **57** (**S@Ag14d**), which encapsulate S^2–^, were reported by Jin and colleagues [124], Fuhr and colleagues [174], and Corrigan and colleagues [156], respectively, and **60** (**Se@Ag14**), which encapsulates Se^2–^, was synthesized by Dehnen and colleagues [175]. Notably, **54** (**S@Ag14a**) and **60** (**Se@Ag14**) are protected by the same ligand, and both have been synthesized using Ag(I)–PR_3_ compounds as an Ag precursor. In the case of **54** (**S@Ag14a**), it was synthesized simply by dissolving the Ag precursor ((PPh_3_)_2_Ag(S_2_CSPh)) in DCM, whereas **60** (**Se@Ag14**) was synthesized by slowly dropping the Se^2–^ precursor (PhSeSiMe_3_) into a THF solution containing the Ag precursor ((PPh_3_)_3_AgNO_3_) and 1,4-bis(trichlorostannyl)butane (Cl_3_Sn(CH_2_)_4_SnCl_3_). Based on these results, it can be inferred that the synthesis of Se@Ag_*n*_ NCs requires more complex steps than that of S@Ag_*n*_ NCs.

**Figure 10.**
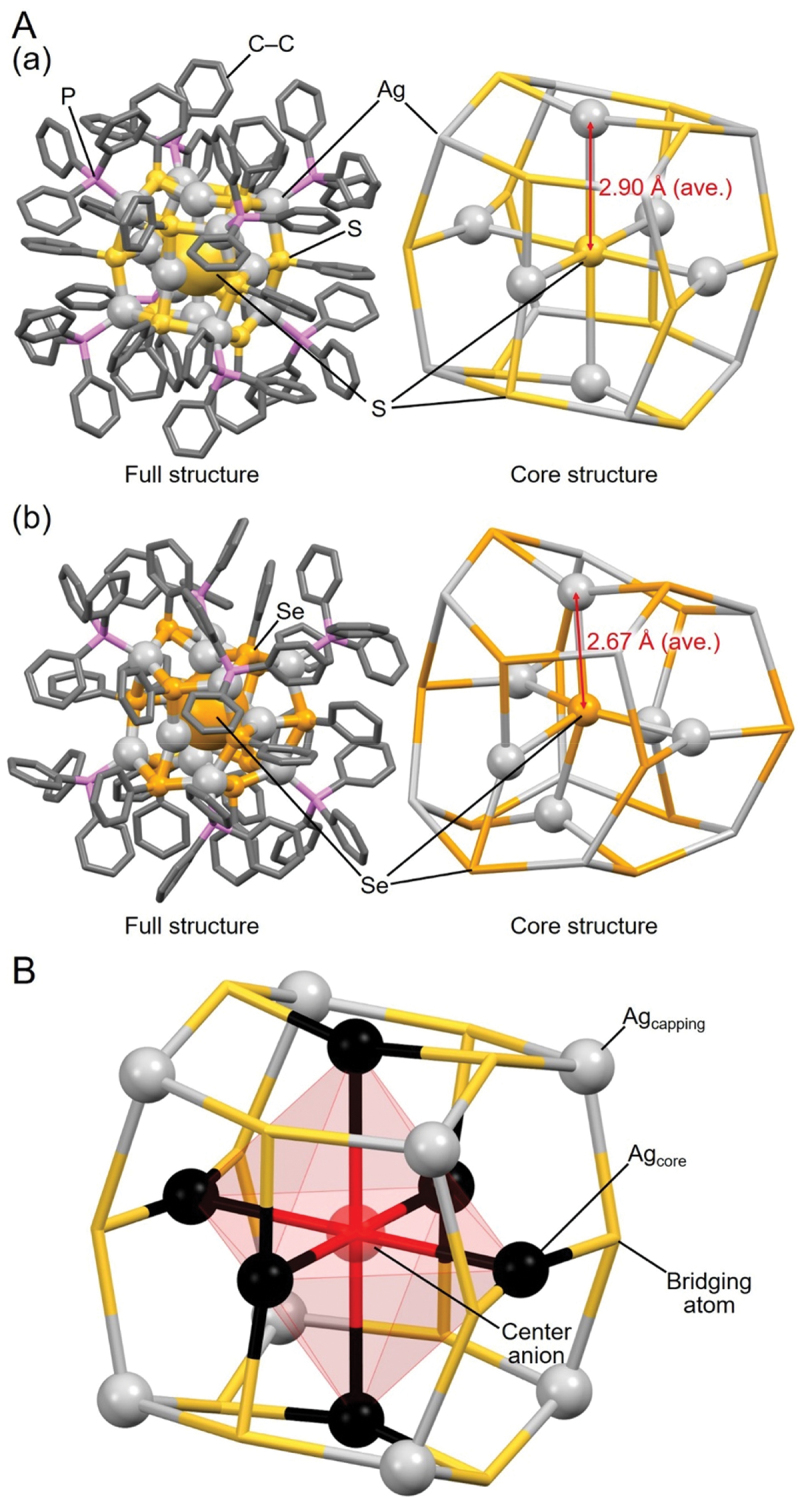
(A) Full (left) and core (right) structures of (a) 54 (**S@Ag14a**) and (b) 60 (**Se@Ag14**). (B) Schematic illustration of the geometric structure in the X’@Ag_14_ NCs model. Data are taken from refs [124,175].

Figure 10A shows the geometric structures of **54** (**S@Ag14a**) and **60** (**Se@Ag14**). Both frameworks have a geometric structure with one Ag atom on each facet of a four-sided bipyramidal core formed by the bonding of the central X’ anion with six Ag atoms (Figure 10B). In these cage structures, consisting of fourteen Ag atoms, chalcogen atoms are bridging the Ag atoms. Thus, these cage structures are different from those of **20** (**F@Ag14**), **21** (**Cl@Ag14a**), **22** (**Cl@Ag14b**), and **23** (**Br@Ag14**), described in Section 3.1.2 (Figure 5). The average X’–Ag distance in **54** (**S@Ag14a**) is 2.91 Å, and that in **60** (**Se@Ag14**) is 2.67 Å. Accordingly, **54** (**S@Ag14a**) has a larger free volume in the cage structure. The longer average X’–Ag bond length in **54** (**S@Ag14a**) indicates that the smaller ionic radii of the central S^2–^ anion (1.91 ± 0.07 Å) [183] is responsible, compared with the larger ionic radii of Se^2–^ (2.09 ± 0.04 Å) [183], which is unlike the X@Ag_*n*_ and X’@Ag_9_ NCs. This probably occurs because the Ag–X’ bond is relatively strong and X’@Ag_14_ NCs cannot easily change their cage structure with the changing ionic radius of the central anion. This also appears to be the reason why the average X’–Ag length in **54** (**S@Ag14a**) is relatively long.

### 3.3. Oxoanion-templated Ag NCs

In the following section, we describe the Ox@Ag_*n*_ NCs that encapsulate Ox ions at the central position. The representative Ox@Ag_*n*_ NCs are summarized in Table 4 [125,135–137,141–143,158,159,172,177,181,184–206]. Among them, we first describe the syntheses and geometric structures of Ag NCs that encapsulate metallic oxide ions (MOx) (Section 3.3.1) or nonmetallic oxide ions (NMOx) (Section 3.3.2). Then, we discuss how the differences in the central anions affect the geometric structures of Ox@Ag_*n*_ NCs (Section 3.3.3).

#### 3.3.1. MOx@Ag_n_ NCs

Typical examples of central MOx anions are chromate (CrO_4_^2–^), molybdate (MoO_4_^2–^), and vanadate (VO_4_^3–^) (Table 4).

**Figure 11.**
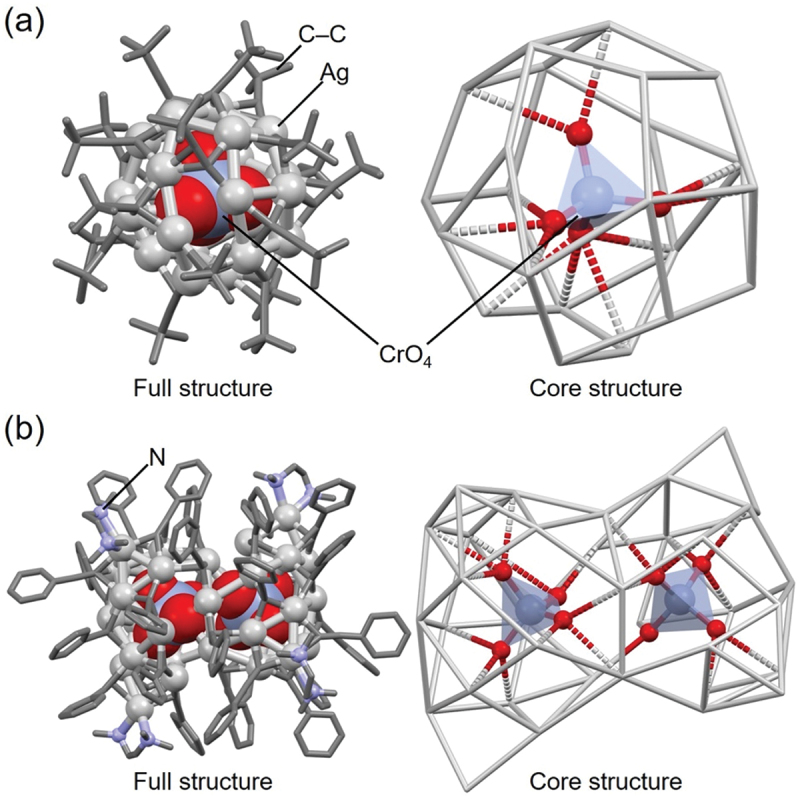
Full (left) and core (right) structures of (a) 118 (**CrO_4_@Ag22**) and (b) 139 (**(CrO_4_)_2_@Ag35**). Data are taken from ref [187].

Pioneering examples for CrO_4_@Ag_*n*_ NCs are **118** (**CrO**_**4**_**@Ag**_**22**_, Figure 11(a)) and **139** (**(CrO**_**4**_)_**2**_**@Ag35**, Figure 11(b)), reported by Wang and colleagues in 2009 [187]. The former, **118** (**CrO**_**4**_**@Ag22**), was synthesized by adding TMEDA to a methanol solution containing [AgC≡C^*t*^Bu]_*n*_ and AgBF_4_ and then adding potassium dichromate (K_2_Cr_2_O_7_). The latter, **139** (**(CrO**_**4**_)_**2**_**@Ag35**, Figure 11(b)), was synthesized using [AgC≡CPh]_*n*_ instead of [AgC≡C^*t*^Bu]_*n*_. In addition to these reports, K_2_Cr_2_O_7_ has often been used as a precursor for CrO_4_^2–^ [136,185,187]. Notably, for **118** (**CrO**_**4**_**@Ag22**), the products did not contain TMEDA. However, even these NCs cannot be synthesized without the addition of TMEDA during synthesis. On the contrary, **139** (**(CrO**_**4**_)_**2**_**@Ag35**) has a geometric structure in which TMEDA is chelated to Ag. Unlike the former, the latter forms a giant peanut-like structure because of the inclusion of two CrO_4_^2–^ anions. Because AgC≡CPh is less bulky than AgC≡C^*t*^Bu, large NCs can form in the synthesis of the latter. These observations agree with those for SR-protected Au_*n*_ NCs [207].

**Figure 12.**
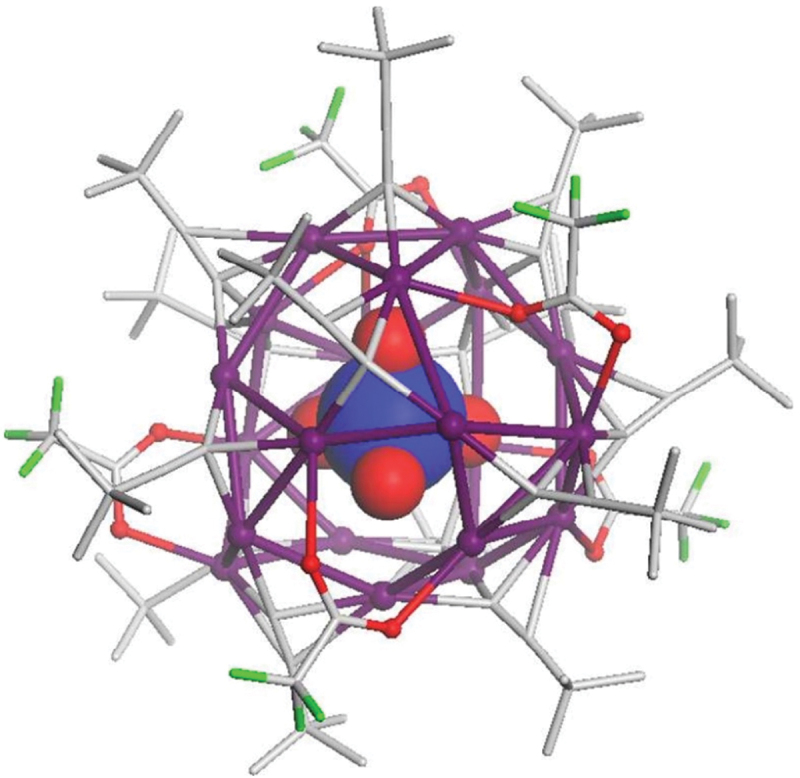
The full geometric structure of 108 (**MoO_4_@Ag20**). Reprinted with permission from [186], copyright (2009, American Chemical Society).

Regarding MoO_4_@Ag_*n*_ NCs, **108** (**MoO**_**4**_**@Ag20**, Figure 12) [186] was synthesized by adding the POM complex [Mo_6_O_19_](^*n*^Bu_4_N)_2_, the precursor of MoO_4_^2–^, and tetraethylammonium hydroxide (Et_4_NOH) to a methanol solution containing AgC≡C^*t*^Bu and AgCF_3_CO_2_ (pH = 6.7, stirring method). For MoO_4_@Ag_*n*_ NCs, unlike the case of CrO_4_@Ag_*n*_ NCs, (NH_4_)_6_Mo_7_O_24_·4 H_2_O, MoO_2_(acac)_2_ (acac = acetylacetone), and Na_2_MoO_4_ can also be used as precursors of the central anion (MoO_4_^2–^) [135,172,191]. When trifluoroacetic acid (CF_3_CO_2_H) was used instead of AgCF_3_CO_2_ to increase the acidity of the solution (pH = 3.5), Ag NCs encapsulating Mo_6_O_19_ (POM) were synthesized instead of MoO_4_^2–^ (MOx). These results indicate that controlling the solution pH is required for synthesizing the MoO_4_@Ag_*n*_ NCs using POM as a precursor [208].

**Figure 13.**
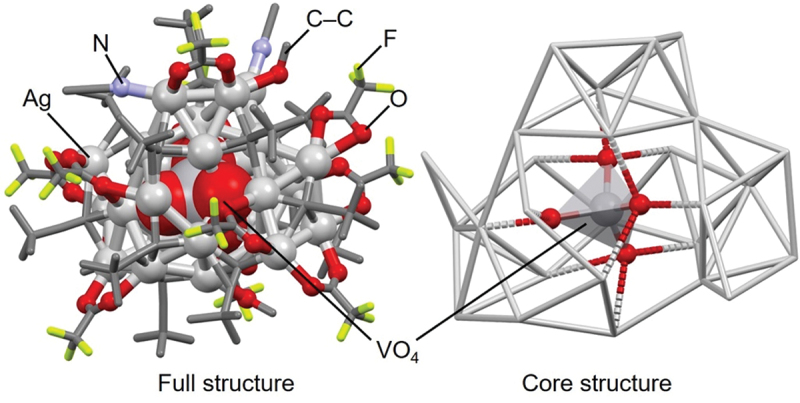
Full (left) and core (right) structures of 132 (**VO_4_@Ag26**). Data are taken from ref [142].

Regarding VO_4_@Ag_*n*_ NCs, **132** (**VO**_**4**_**@Ag26**, Figure 13) was synthesized by adding ammonium metavanadate (NH_4_VO_3_) to a MeOH/MeCN mixture containing [AgC≡C^*t*^Bu]_*n*_ and AgCF_3_CO_2_ and heating the solution to 70 °C (solvothermal method). It has been experimentally shown that larger Ag NCs can be formed when trivalent VO_4_^3–^ is used as a template source than when divalent CrO_4_^2–^ and MoO_4_^2–^ are used, although the reason remains unclear.

#### 3.3.2. NMOx@Ag_n_ NCs

Anions in the form of NMOx include CO_3_^2–^, NO_3_^–^, X’O_3_^2–^, X’O_4_^2–^ (X’ = S, Se, Te), and ClO_4_^–^. Representative NMOx@Ag_*n*_ NCs are described in Table 4. Salts containing the respective oxides are generally used as precursors for the central NMOx anion. However, there are exceptions. For example, in the synthesis of **91** (**CO**_**3**_**@Ag17**) and **94** (**CO**_**3**_**@Ag19**) [125], the central anion was produced by the conversion of atmospheric CO_2_ to CO_3_^2–^ by TMEDA. The use of atmospheric CO_2_ was also adapted in the synthesis of **99** (**CO**_**3**_**@Ag20a**) and **100** (**CO**_**3**_**@Ag20b**).

**Figure 14.**
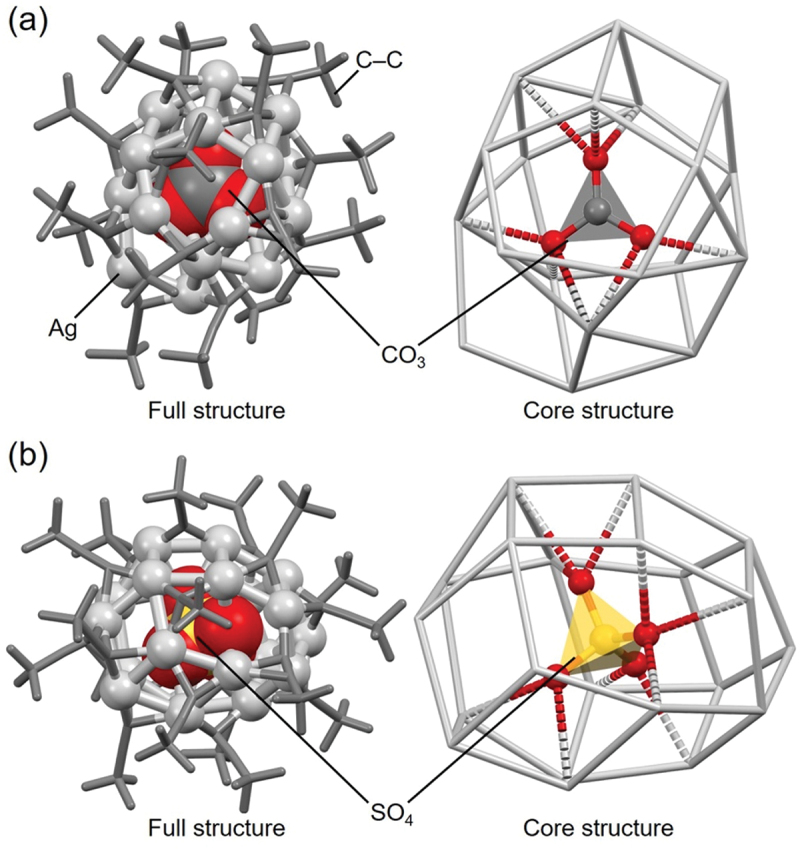
Full (left) and core (right) structures of (a) 94 (**CO_3_@Ag19**) and (b) 113 (**SO_4_@Ag21**). Data are taken from refs [125,187].

The geometric structures of **94** (**CO**_**3**_**@Ag19**) [125] and **113** (**SO**_**4**_**@Ag21**) [187], protected by the same ligand, are shown in Figure 14. Both NMOx@Ag_*n*_ NCs were reported by Wang and colleagues in 2009 and were synthesized using similar compounds and methods, except for the central anion precursor. Despite the similarities in their syntheses, these NMOx@Ag_*n*_ NCs differ in the number of constituent Ag atoms, with **113** (**SO**_**4**_**@Ag21**) exhibiting a larger Ag cage structure than **94** (**CO**_**3**_**@Ag19**). The NMOx anions encapsulated in **94** (**CO**_**3**_**@Ag19**) and **113** (**SO**_**4**_**@Ag21**) contain three and four O atoms, respectively. Because Ag atoms form bonds with O atoms when NMOx acts as a central anion, SO_4_^2–^ can form bonds with a larger number of Ag atoms than CO_3_^2–^. The SO_4_^2–^ also requires a larger ionic volume than CO_3_^2–^. These factors contribute to the formation of Ag cage structures that are larger in SO_4_@Ag_*n*_ NCs than in CO_3_@Ag_*n*_ NCs.

#### 3.3.3. Comparison between Ox@Agn NCs

In 2014, Liu and colleagues reported the synthesis of **87** (**[SO**_**4**_**@Ag16]**_**2**_), **88 ([SeO**_**4**_**@Ag16]**_**2**_), **89** (**[CrO**_**4**_**@Ag16]**_**2**_), and **90** (**[MoO**_**4**_**@Ag16]**_**2**_). Although these Ox@Ag_*n*_ NCs include the same ligand, they have different central Ox anions.

**Figure 15.**
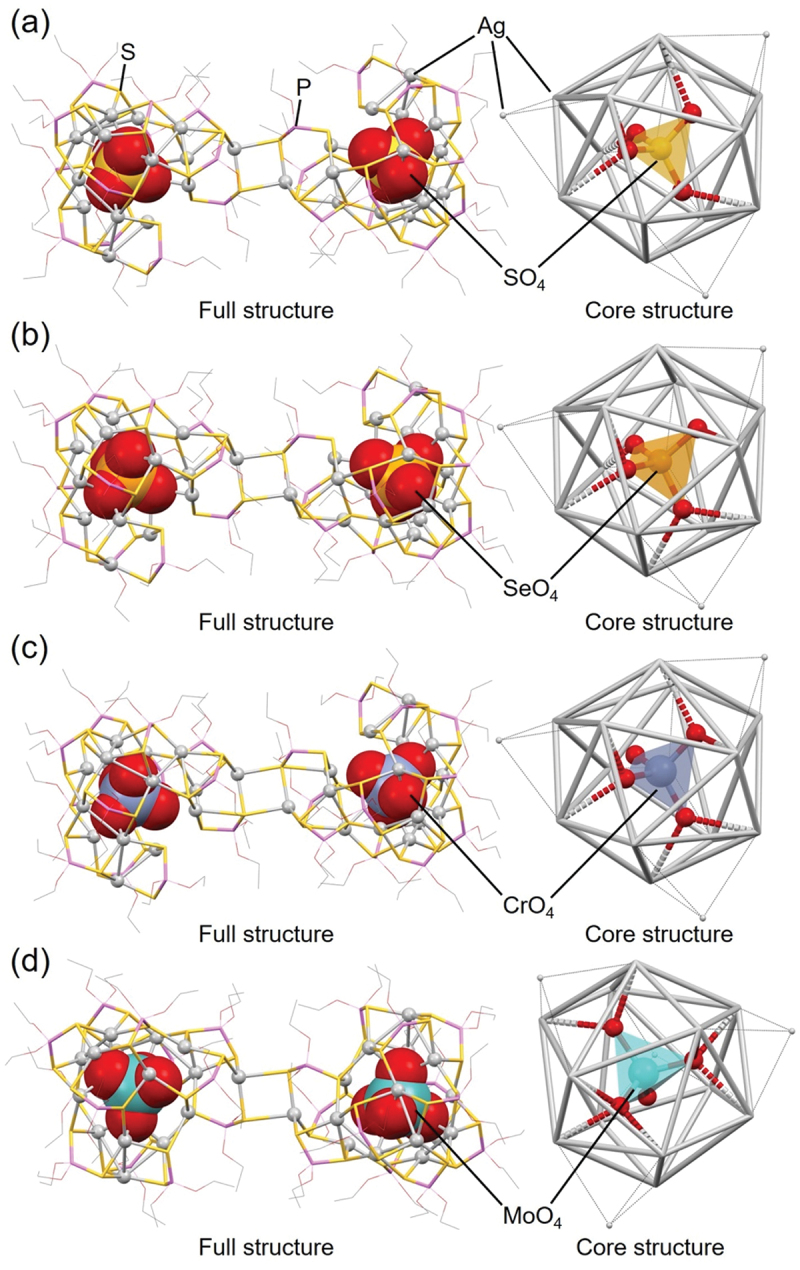
Full (left) and core (right) structures of (a) 87 (**[SO_4_@Ag16]_2_**), (b) 88 (**[SeO_4_@Ag16]_2_**), (c) 89 (**[CrO_4_@Ag16]_2_**), and (d) 90 (**[MoO_4_@Ag16]_2_**). Data are taken from ref [191].

Figure 15 shows the geometric structures of **87** (**[SO**_**4**_**@Ag16]**_**2**_), **88** (**[SeO**_**4**_**@Ag16]**_**2**_), **89** (**[CrO**_**4**_**@Ag16]**_**2**_), and **90** (**[MoO**_**4**_**@Ag16]**_**2**_) [191]. These Ox@Ag_*n*_ NCs have similar Ag cage structures, and Ox is surrounded by sixteen Ag atoms in all of them, which consists of an icosahedral Ag_12_ framework with four surrounding Ag atoms. The structures of these Ox@Ag_*n*_ NCs are dimerized via S and Ag bonding.

However, several differences also exist in the coordination mode between the central Ox anion and Ag atoms, and in the degree of distortion in the Ag cage structures (Figure 15). Specifically, differences occur in the Ag–O coordination mode, with *µ*_4_; *η*^2^, *η*^1^, *η*^1^ for **87** (**[SO**_**4**_**@Ag16]**_**2**_), *µ*_6_; *η*^2^, *η*^2^, *η*^1^, *η*^1^ for **88** (**[SeO**_**4**_**@Ag16]**_**2**_), *µ*_6_; *η*^2^, *η*^2^, *η*^2^ for **89** (**[CrO**_**4**_**@Ag16]**_**2**_), and *µ*_8_; *η*^3^, *η*^3^, *η*^2^ for **90** (**[MoO**_**4**_**@Ag16]**_**2**_). In addition, **87** (**[SO**_**4**_**@Ag16]**_**2**_), **88** (**[SeO**_**4**_**@Ag16]**_**2**_), and **89** (**[CrO**_**4**_**@Ag16]**_**2**_) have a uniform orientation of tetrahedral Ox against the Ag framework, whereas the orientation of tetrahedral Ox in **90** (**[MoO**_**4**_**@Ag16]**_**2**_) is different (Figure 15).

The sizes of the central anions also differ slightly among **87** ([**SO**_**4**_**@Ag16]**_**2**_), **88** (**[SeO**_**4**_**@Ag16]**_**2**_), and **89 ([CrO**_**4**_**@Ag16]**_**2**_), which affects their overall structure. For example, the average Ag–Ag distances in the icosahedral Ag_12_ framework surrounding the central anion Ox are estimated to be 4.016 Å, 4.051 Å, and 4.033 Å, respectively. The order of the average Ag–Ag distances is consistent with that of the average central atom–O distances in the central Ox anion (SO_4_^2–^, 1.464 Å; SeO_4_^2–^, 1.632 Å; CrO_4_^2–^, 1.628 Å). Thus, the size of the Ag framework in Ox@Ag_*n*_ NCs depends on the size of the central Ox anion.

For **90** (**[MoO**_**4**_**@Ag16]**_**2**_), the average Mo–O distance for the central MoO_4_^2–^ anion is 1.721 Å, which is longer than the central atom–O distance in the other Ox systems. This may be the reason why **90** (**[MoO**_**4**_**@Ag16]**_**2**_) encapsulates the central MoO_4_^2–^ anion in a different orientation compared with the other Ox@Ag_*n*_ NCs. The average Ag–Ag distance (4.052 Å) in the icosahedral Ag_12_ framework is also longer in **90** (**[MoO**_**4**_**@Ag16]**_**2**_) than in **87** (**[SO**_**4**_**@Ag16]**_**2**_), **88** (**[SeO**_**4**_**@Ag16]**_**2**_), and **89** (**[CrO**_**4**_**@Ag16]**_**2**_). However, the degree of extension in the icosahedral Ag_12_ framework in **90** (**[MoO**_**4**_**@Ag16]**_**2**_) is not significant (0.001 Å) when accounting for the difference in size between SeO_4_^2–^ and MoO_4_^2–^ anions (0.089 Å). In **90** (**[MoO**_**4**_**@Ag16]**_**2**_), the central MoO_4_^2–^ anion is encapsulated in a different orientation compared with the others, which appears to induce a relaxation of the extension in the icosahedral Ag_12_ framework.

**Figure 16.**
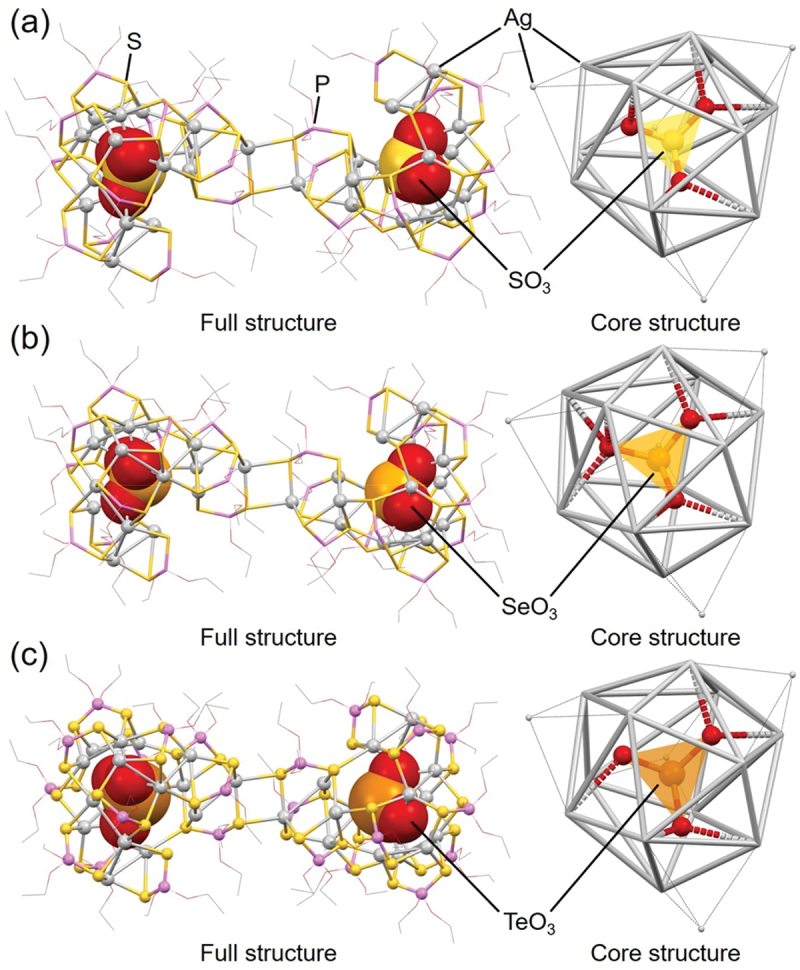
Full (left) structures and core (right) structures of (a) 82 (**[SO_3_@Ag16]_2_**), (b) 83 (**[SeO_3_@Ag16]_2_**), and (c) 84 (**[TeO_3_@Ag16]_2_**). Data are taken from ref [192].

In addition, **82** (**[SO**_**3**_**@Ag16]**_**2**_), **83** (**[SeO**_**3**_**@Ag16]**_**2**_), and **84** (**[TeO**_**3**_**@Ag16]**_**2**_) were reported by Liu and colleagues in 2015 (Table 4) [192]. The geometric structures of these Ox@Ag_*n*_ NCs (Figure 16) are similar to **87** (**[SO**_**4**_**@Ag16]**_**2**_) and **88** (**[SeO**_**4**_**@Ag16]**_**2**_) (Figure 15). In Section 3.3.2, we explained that the central Ox anion can induce different geometric structures (e.g. planar triangular or tetrahedral) depending on the number of O atoms, and Ox@Ag_*n*_ NCs with different numbers of Ag atoms can be formed depending on the central Ox anion. However, **82** (**[SO**_**3**_**@Ag16]**_**2**_), **83** (**[SeO**_**3**_**@Ag16]**_**2**_), and **84** (**[TeO**_**3**_**@Ag16]**_**2**_) have the same number of Ag atoms and the same shape, despite having a different number of O atoms in the central Ox anion compared with **87** (**[SO**_**4**_**@Ag16]**_**2**_) and **88** (**[SeO**_**4**_**@Ag16]**_**2**_). This is likely because the central X’O_3_^2–^ anion (X’ = S, Se, or Te) has a triangular pyramidal shape, instead of a triangular shape (Figure 16). In **82** (**[SO**_**3**_**@Ag16]**_**2**_), **83** (**[SeO**_**3**_**@Ag16]**_**2**_), and **84** (**[TeO**_**3**_**@Ag16]**_**2**_), there are only three O atoms bonded to the triangular pyramid, but there are lone pairs at the apex (X’) of the pyramid, which maintains the surrounding icosahedral Ag_12_ framework. These results indicate that the geometric structure of Ox@Ag_*n*_ NCs is greatly affected not only by the number of O atoms in the Ox anion but also by the shape of the Ox anion.

### 3.4. Polyoxometalate-templated Ag NCs

Table 5 summarizes the representative POM@Ag_*n*_ NCs [135,137–140,152,158,159,184,186,202,204,209–228]. Many of them contain polyoxovanadate (POV@Ag_*n*_ NCs), polyoxomolybdate (POMo@Ag_*n*_ NCs), and polyoxotungstate (POW@Ag_*n*_ NCs), and the number of reported examples decreases in the order of POMo@Ag_*n*_ NCs > POV@Ag_*n*_ NCs > POW@Ag_*n*_ NCs. More than half of the POM@Ag_*n*_ NCs were reported after 2018, demonstrating that these anion-templated Ag_*n*_ NCs have been receiving attention more recently.

The POM@Ag_*n*_ NCs are characterized by a higher number of constituent Ag atoms than other anion-templated Ag_*n*_ NCs, such as X@Ag_*n*_ NCs, X’@Ag_*n*_ NCs, and Ox@Ag_*n*_ NCs. This is because the encapsulated POMs are generally larger than X, X’, and Ox. The smallest POM@Ag_*n*_ NC reported is **148** (**POV@Ag22**) [204], which encapsulates [V_2_O_7_]^4–^ and was found in 2022 by Jin and colleagues. Despite having the smallest size, **148** (**POV@Ag22**) is larger than most Ag NCs that encapsulate X or X’. Therefore, POM is effective in synthesizing Ag NCs with somewhat larger frameworks. The history of research on POM@Ag_*n*_ NCs is short, and currently, the largest anion-templated Ag NCs are **39** (**Cl@Ag216**) [155] and **147** (**SO**_**4**_**@Ag78**) [189]. However, it is expected that larger anion-templated Ag NCs will be created in the future using POM as an anion template.

Mak and colleagues first reported on **149** (**POV@Ag24a**), **165** (**POV@Ag40a**), and **167** (**POMo@Ag40**) in 2009 [186]. POM had already attracted attention as a functional material at that time, and because the O atom of POM has a high affinity for Ag(I) ions, there had been several reports on the synthesis of POM–Ag(I) complexes before then [229–234]. However, there were no reported examples of compounds that encapsulate POM in the Ag framework until the report of Mak and colleagues.

**Figure 17.**
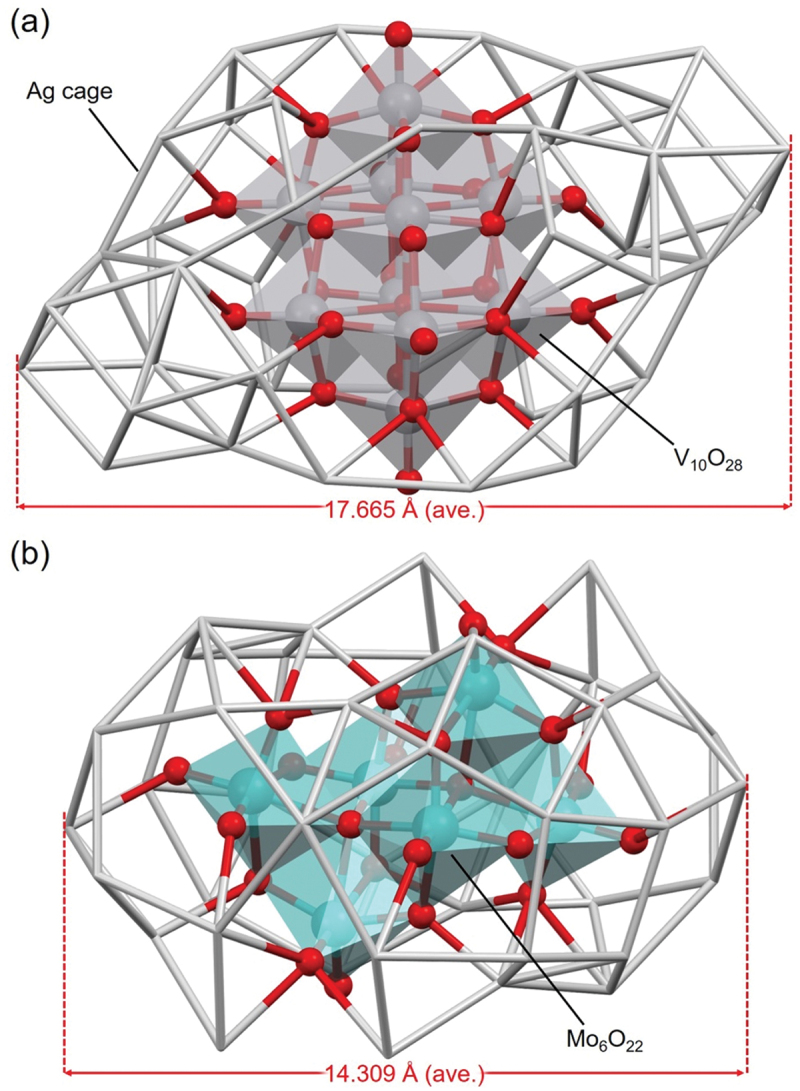
The full geometric structures of (a) 165 (**Pov@Ag40a**), and (b) 167 (**POMo@Ag40**). Data are taken from ref [186].

In that report, they also found that the chemical compositions of the products vary depending on the presence or absence of stabilizers in the organic solvent during the reaction. Specifically, they compared the chemical composition of the products when CF_3_CO_2_H was added as a stabilizer for POM to make the solution acidic and when AgCF_3_CO_2_ was added as a stabilizer for POM to make the solution neutral. In the former experimental condition, **165** (**POV@Ag40a**) or **167** (**POMo@Ag40**) was formed, encapsulating [V_10_O_28_]^6–^ or [Mo_6_O_22_]^8–^, whereas in the latter experimental condition, **149** (**POV@Ag24a**) or **108** (**MoO**_**4**_**@Ag20**) was formed, encapsulating smaller POMs such as [V_2_O_7_]^4–^ or Ox, [MoO_4_]^2–^ (Table 4). Regarding **165** (**POV@Ag40a**) and **167** (**POMo@Ag40**), although they contain the same number of Ag atoms, there is a large difference in the edge-to-edge Ag–Ag distances between them (17.665 Å for **165** (**POV@ Ag40a**) vs. 14.309 Å for **167** (**POMo@Ag40**), Figure 17). This is because the Ag framework in **167** (**POMo@Ag40**) is regularly and compactly surrounding the central [Mo_6_O_22_]^8–^ anion, resulting in a more spherical shape compared with that of **165** (**POV@Ag40a**).

Many studies on the synthesis of novel POM@Ag_*n*_ NCs and their mechanisms have been reported since 2009. Reviews of these POM@Ag_*n*_ NCs have recently been reported by Li, Zheng, and colleagues [127], and Hu, Shi, Ji, and colleagues [129]. Therefore, we look at the differences between POM@Ag_*n*_ NCs and other anion-templated Ag NCs, which have not been described in previous reviews.

**Figure 18.**
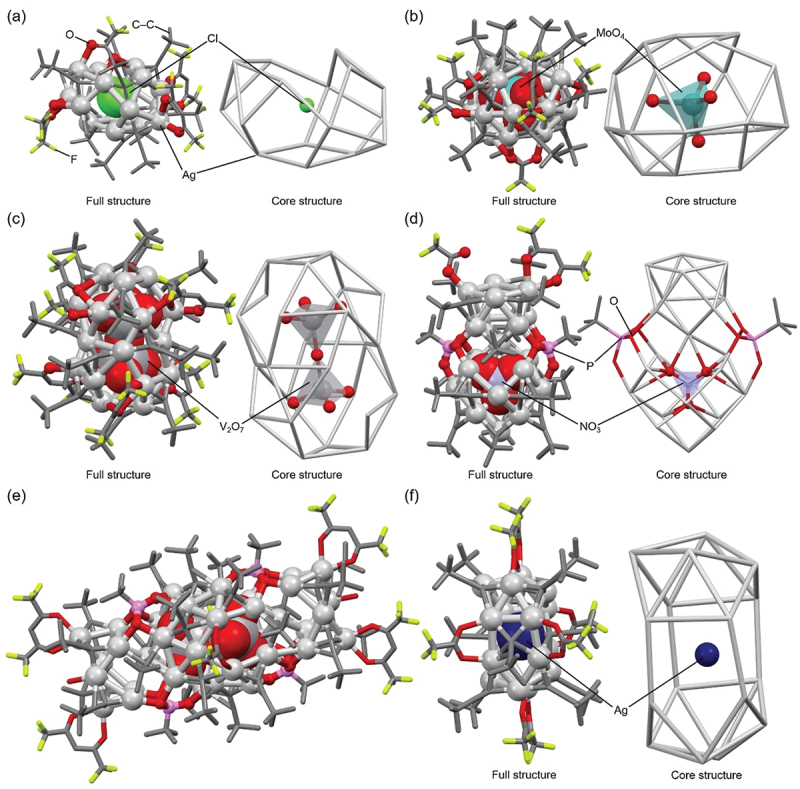
Full (left) and core (right) structures of (a) 29 (**Cl@Ag16c**), (b) 98 (**MoO_4_@Ag19**), (c) 150 (**POV@Ag24b**), (d) 117 (**NO_3_@Ag22**), (e) 166 (**POV@Ag40b**) and (f) **Ag@Ag16**. For (a)–(d) and (f), the core structure is shown to the right of the full structure, respectively. Data are taken from ref [159].

For example, **29** (**Cl@Ag16c**, Table 2, Figure 18(a)), **98** (**MoO**_**4**_**@Ag19**, Table 4, Figure 18(b)), and **150** (**POV@Ag24b**, Table 5, Figure 18(c)) have increasing numbers of Ag atoms in this order [159]. This result demonstrates the trend described at the beginning of this section (Section 3.4), stating that POM encapsulation tends to yield larger-sized Ag NCs. This trend is also observed in the Ag NCs linked by phosphonic acid in the Ag framework (**117** (**NO**_**3**_**@Ag22**, Figure 18(d)) and **166** (**POV@Ag40b**, figure 18(e))). In addition, Jin and colleagues reported the synthesis of Ag NCs (**Ag@Ag16**, Figure 18(f)) in which the central Cl^−^ anion in **29** (**Cl@Ag16c**) is replaced by an Ag atom [159]. **Ag@Ag16** has the same number of Ag atoms, but a different cage structure, compared with **29** (**Cl@Ag16c**). This result indicates that Ag NCs without the central anion can be synthesized by making slight modifications to the synthesis methods of anion-templated Ag NCs described in this review.

### 3.5. Hydride/deuteride-templated Ag NCs

Finally, we describe Ag NCs that contain H^−^ and D^−^ as central anions. Table 6 summarizes the representative H/D@Ag_*n*_ NCs [132,235–240]. The number of reports on H/D@Ag_*n*_ NCs is smaller than that on other anion-templated Ag NCs, and the first report of H/D@Ag_*n*_ NCs was in 2010. In H/D@Ag_*n*_ NCs, the Ag_11_ framework has the highest number of constituent Ag atoms. Thus, H and D tend to yield smaller Ag NCs than those obtained with other anions.

The earliest H/D@Ag_*n*_ NCs were **207** (**H@Ag8a**), **208** (**H@Ag8b**), **209** (**H@Ag8c**), **212** (**D@Ag8a**), **213** (**D@Ag8b**), and **214** (**D@Ag8c**), all reported in 2010 by Liu and colleagues [132,236]. Although the formation of [Ag_4_H]^+^ complexes had been previously observed in the gas phase [241], the isolation of ligand-protected H/D@Ag_*n*_ NCs had not been reported. Regarding the syntheses, **207** (**H@Ag8a**) and **208** (**H@Ag8b**) were obtained by stirring Ag(MeCN)_4_PF_6_ and NH_4_Se_2_P(OR)_2_ (*R* = ^*i*^Pr or Et) in THF for 1 h, then cooling the resulting solution to −20 °C, to which NaBH_4_ was added. Similarly, **212** (**D@Ag8a**) and **213** (**D@Ag8b**) were obtained, but using NaBD_4_ instead of NaBH_4_. The synthesis of **209** (**H@Ag8c**) and **214** (**D@Ag8c**) was conducted in a similar manner. Interestingly, these synthesis methods are similar to those of H-protected Ag nanoclusters [242,243], but the products were different. This reason is expected to be clarified in the future work. In addition to such direct synthesis methods, they also added NaBH_4_ to solutions of X@Ag_8_ NCs (**2** (**F@Ag8a**) or **7** (**Cl@Ag8d**), table 2) and monitored the reaction process by ^31^P NMR spectroscopy. In both cases, the central anion was replaced by H and D within a few minutes, inducing the formation of **209** (**H@Ag8c**) and **214** (**D@Ag8c**).

**Figure 19.**
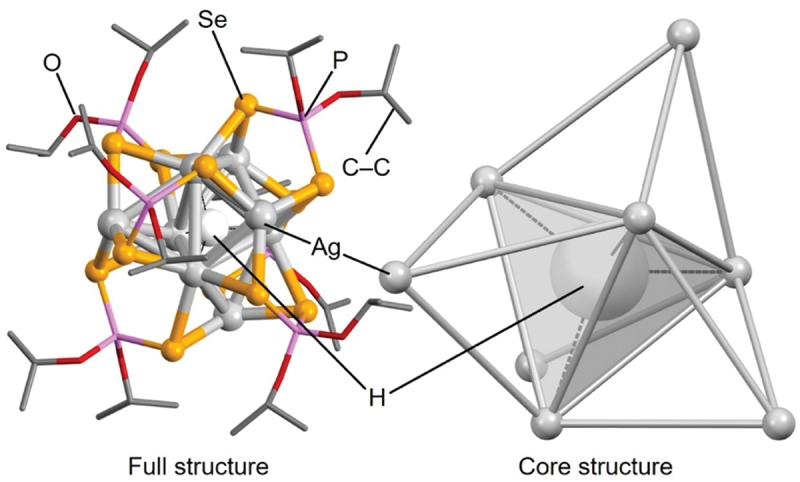
Full (left) and core (right) structures of 207 (**H@Ag8a**). Data are taken from ref [236].

As an example of the H/D@Ag_8_ NCs, Figure 19 shows the geometric structure of **207** (**H@Ag8a**) [236]. The Ag atoms that form the Ag framework are classified into two categories: 1) Ag atoms that form a tetrahedron surrounding H^−^ and 2) Ag atoms that surround the tetrahedron. Thus, the Ag_8_ framework is formed by four Ag atoms capping each triangular facet of a tetrahedron composed of four Ag, which is different from the distorted cubic framework of X@Ag_8_ NCs (Figure 3) [132].

**Figure 20.**
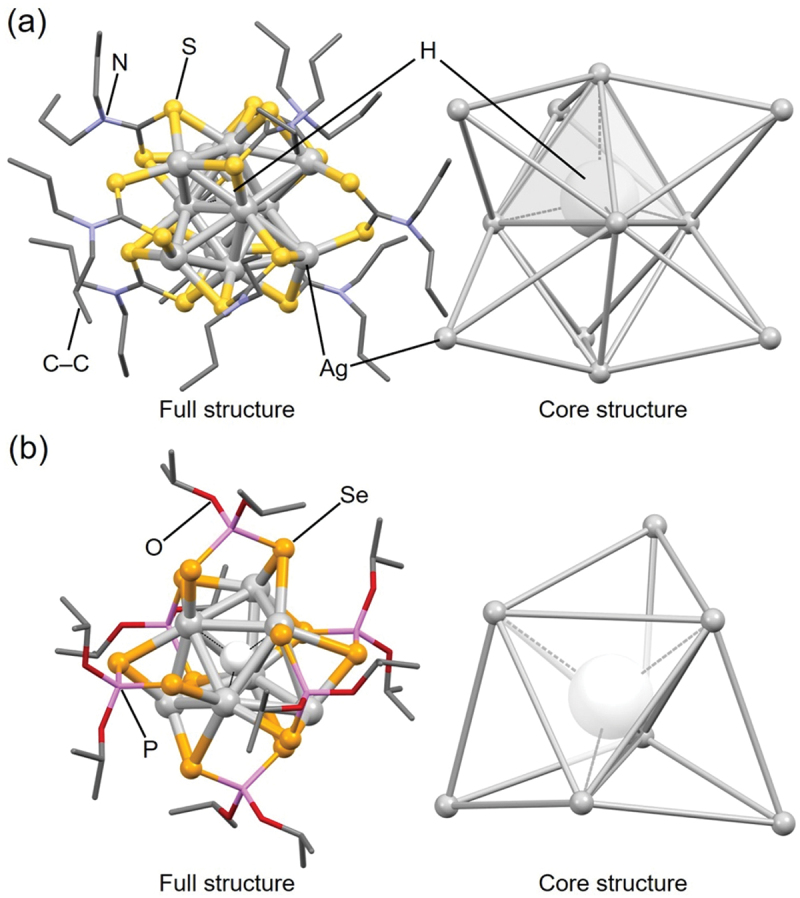
Full (left) and core (right) structures of (a) 216 (**H@Ag11**) and (b) 201 (**H@Ag7a**). Data are taken from refs [235,237].

**Figure 21.**
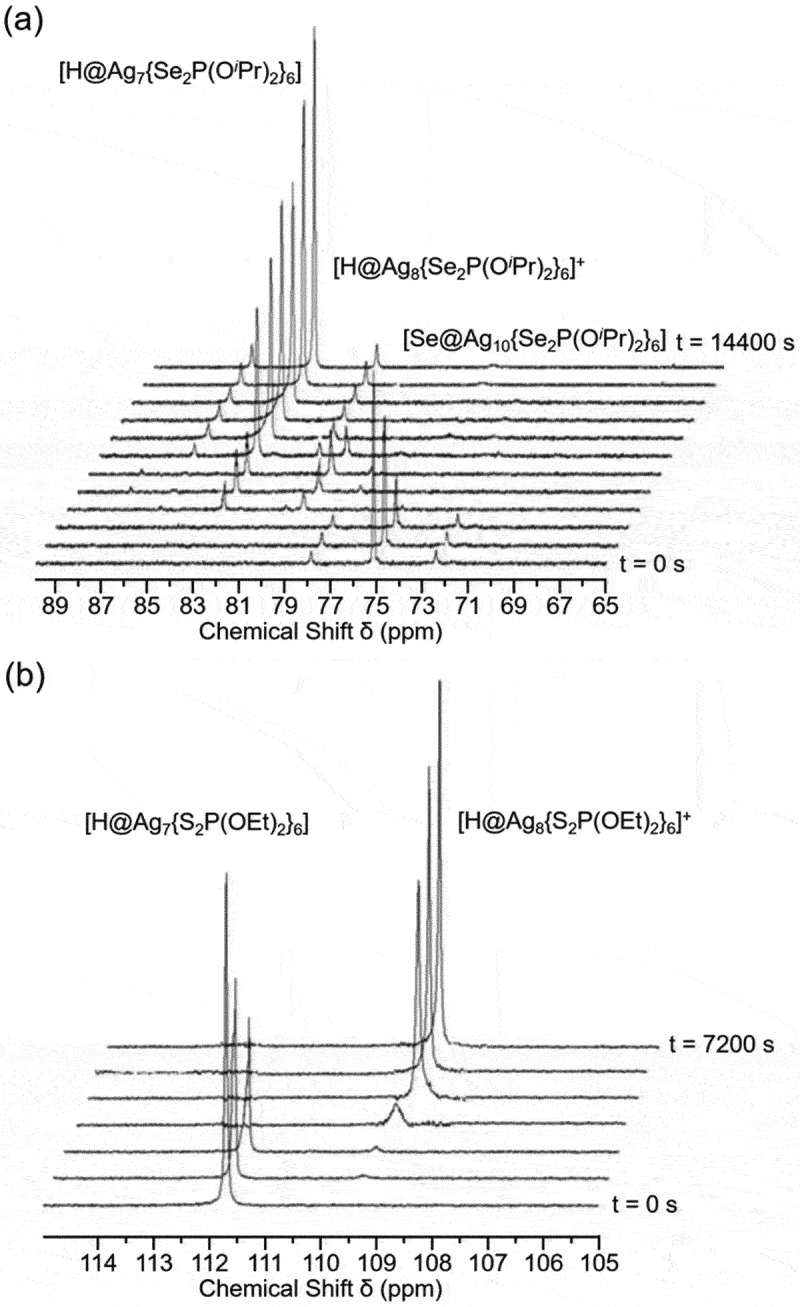
(A) the transformation of Se@Ag_10_ to H@Ag_8_ to H@Ag_7_. (b) Silver uptake of [H@Ag_7_{S_2_P(OEt)_2_}_6_] to [H@Ag_8_{S_2_P(OEt)_2_}_6_]^+^ in the presence of one equivalent of [Ag(MeCN)_4_][PF_6_] (fig7, 21), monitored by ^31^P NMR spectroscopy. Reprinted with permission from [235], copyright (2013, American Chemical Society).

Liu and colleagues also reported other H/D@Ag_*n*_ NCs, including H/D@Ag_7_ NCs and H/D@Ag_11_ NCs. For example, **216** (**H@Ag11**, Figure 20(a)) and **217** (**D@Ag11**) were reported in 2011 [237], and **201** (**H@Ag7a**, Figure 20(b)), **202** (**H@Ag7b**), **204** (**D@Ag7a**), and **205** (**D@Ag7b**) were reported in 2013 [235]. Overall, the synthetic methods are similar to those for H/D@Ag_8_ NCs. Specifically, for **216** (**H@Ag11**) and **217** (**D@Ag11**), AgNO_3_ was used as the Ag salt and NaS_2_CNPr_2_ as the ligand precursor, which were mixed according to the stoichiometric ratio, and the resulting solutions were then reduced in the liquid phase at −20 °C for 3 hours. For **201** (**H@Ag7a**), **202** (**H@Ag7b**), **204** (**D@Ag7a**), and **205** (**D@Ag7b**), Ag(CH_3_CN)_4_PF_6_ was used as the Ag salt and NH_4_Se_2_P(O^*i*^Pr)_2_ or NH_4_S_2_P(OEt)_2_ as the ligand precursor. The resulting DCM solution was reduced with NaBH_4_ or NaBD_4_ at room temperature to obtain the H/D@Ag_7_ NCs. H@Ag_7_ NCs can also be synthesized by the reaction of X’@Ag_10_(E_2_P(OR)_2_)_8_ (X’ = Se, *R* = ^*i*^Pr or X’ = S, *R* = Et) with two equivalent amounts of [BH_4_]^–^. Reaction tracking by ^31^P NMR revealed that 1) H@Ag_8_ NCs, such as **207** (**H@Ag8a**), are formed as reaction intermediates in these reactions (Figure 21(a)), and 2) H@Ag_8_ NCs are obtained (regenerated) by the reaction of the obtained H@Ag_7_ NCs with an equal amount of Ag salt (Figure 21(b)).

Figure 20(a) shows the geometric structure of **216** (**H@Ag11**). The Ag_11_ framework is formed by capping six Ag atoms against the three-way bipyramidal Ag_5_ structure, with the central H^−^ anion encapsulated in only one of the triangular pyramids. Because the H is difficult to observe by SC-XRD, the location of the H is concluded by predicting the geometric structure with the minimum energy using density functional theory calculations. This is the first example of such a three-way bipyramidal molecular structure capped by six Ag atoms [237].

In **201** (**H@Ag7a**, Figure 20(b)), H is encapsulated in the tetrahedral Ag_4_ framework, and three of the four triangles in the tetrahedron are capped by Ag atoms to form the Ag_7_ framework. The geometric structure of **201** (**H@Ag7a**) is the same as that of **207** (**H@Ag8a**), except one capped Ag atom is removed from the Ag_8_ framework. The difference in the number of Ag atoms in both cases leads to a difference in the coordination between the Ag(I) and ligand. For example, in **207** (**H@Ag8a**), one coordination mode (*μ*_2_, *μ*_2_) occurs between Ag and the two Se atoms in Se_2_P(O^*i*^Pr)_2_, whereas in **201** (**H@Ag7a**), two coordination modes (*μ*_2_, *μ*_2_ and *μ*_2_, *μ*_1_) occur between Ag and the two Se atoms. Removing one capped Ag atom cleaves the three Ag–Se bonds. This results in a new coordination mode (*μ*_2_, *μ*_1_) between the three Se and Ag atoms, changing the coordination pattern between the Ag(I) and ligand [235].

**Figure 22.**
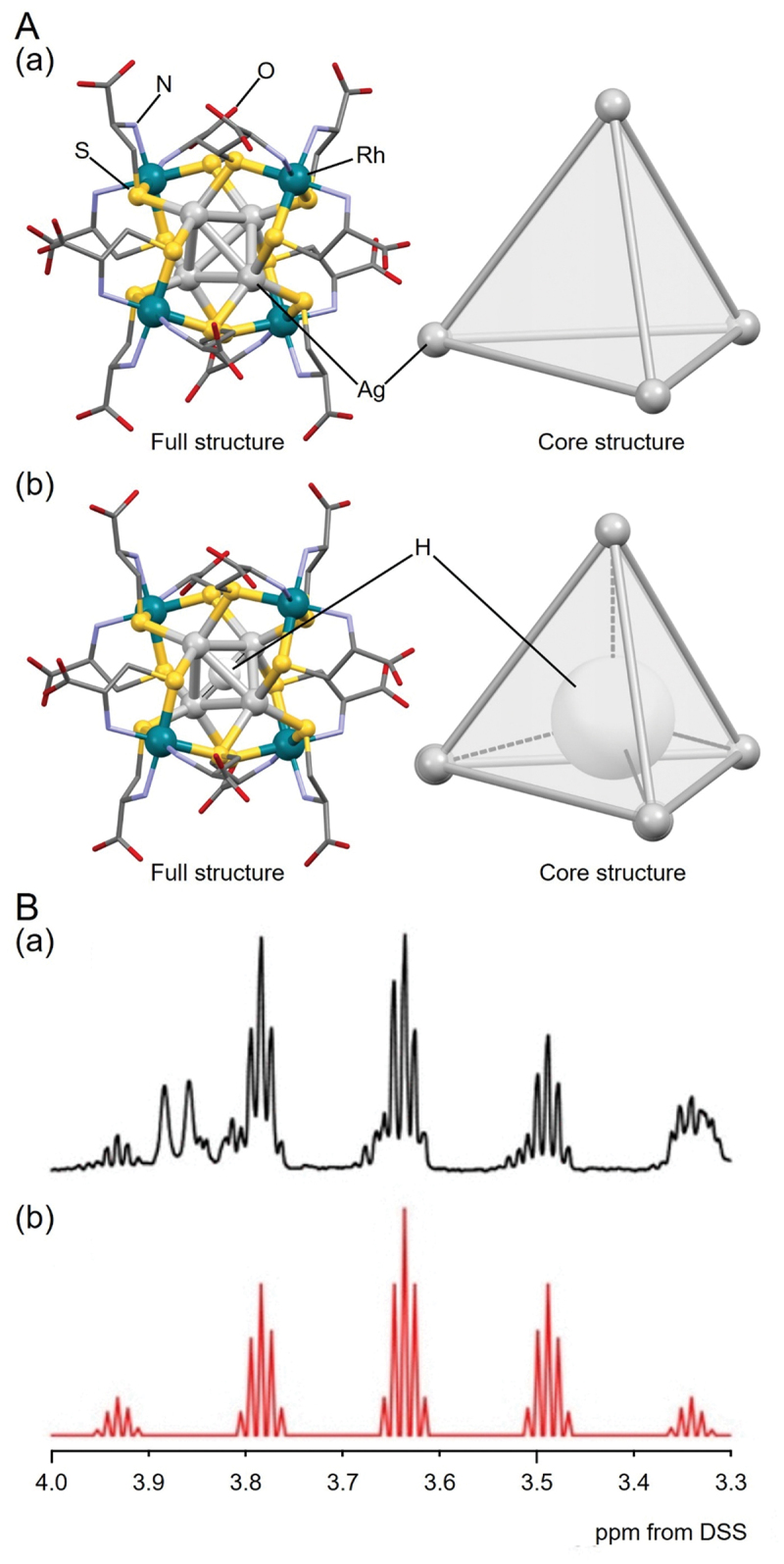
(A) Full (left) and core (right) structures of (a) Ag4Rh4 and (b) 199 (**H@Ag4Rh4**). (B) (a) Observed ^1^H NMR spectrum of 199 (**H@Ag4Rh4**) in NaOH/D_2_O and (b) simulated ^1^H NMR spectrum for the [Ag_4_H]^3+^ moiety. Sodium 4,4-dimethyl-4-silaphentane-1-sulfonate (DSS) was used as the internal reference. Data are taken from ref [240]. Reprinted with permission from [240], copyright (2021, American Chemical Society).

In the H/D@Ag_*n*_ NCs described above, the H/D@Ag_4_ core structure is included. For a long time, there has been no report on the isolation of H/D@Ag_4_ NCs without capping by Ag atoms. In 2021, Konno and colleagues succeeded in isolating two types of H/D@Ag_4_ NCs, **199** (**H@Ag4Rh4**) and **200** (**D@Ag4Rh4**) [240]. Their synthesis methods differ significantly from the syntheses of H/D@Ag_*n*_ NCs described above. Specifically, **199** (**H@Ag4Rh4**) and **200** (**D@Ag4Rh4**) were formed by inserting H or D into [Ag_4_{Rh(L-Cys)_3_}_4_][Na]_8_ (**Ag4Rh4**, Figure 22A(a)), which has an empty Ag framework. They reacted **Ag4Rh4** with NaBH_4_ or NaBD_4_ in an aqueous NaOH solution, followed by the addition of an excess amount of ethanol to obtain powders of **199** (**H@Ag4Rh4**, Figure 22A(b)) or **200** (**D@Ag4Rh4**). They also examined the reaction mechanism for this reaction by X-ray absorption spectroscopy and magnetic measurements. The results suggested that this reaction did not cause the reduction of Ag(I) in [Ag(I)_4_]^4+^ by NaBH_4_ but caused the formation of [Ag(I)_4_H]^3+^, in which H is included at the central position. Because it is difficult to definitively conclude the position of H in the product using SC-XRD, they confirmed the presence and position of the central H anion by ^1^H NMR analysis (Figure 22B). The overall geometric structures of **Ag4Rh4** and **199** (**H@Ag4Rh4**) are similar, both having a slightly distorted tetrahedral Ag_4_ framework. These geometric structures are also similar to (Δ)4-[Zn_4_O(Rh(L-Cys)_3_)_4_] [244–247]. However, closer inspection reveals that the structure is slightly more contracted in **199** (**H@Ag4Rh4**) than that in **Ag4Rh4**, which indicates that the insertion of H may contract the structure of the Ag NCs. The contraction of the framework is also observed in Cu_8_ NCs with H^−^ inclusion [247, 248].

For **199** (**H@Ag4Rh4**, $J_{H{-}^{107}Ag}$ = 68.75 Hz, $J_{H{-}^{108}Ag}$ = 79.25 Hz), it has also been shown that the coupling constant between the Ag atom and central H^−^ anion is approximately twice as large as that of **207** (**H@Ag8a**, $J_{Ag-H}$ = 33 Hz). The higher coupling constant is explained by the average bond distance between the Ag atom and central H^−^ anion, which is much shorter in **199** (**H@Ag4Rh4**) (1.86 Å) compared with that in **207** (**H@Ag8a**) (2.50 Å).

## 4. Conclusion

This review summarizes the synthesis and geometric structure of a wide variety of anion-templated Ag NCs. Through this summary, the following points were discussed:

1. The current synthesis methods are broadly categorized into the stirring method, solvothermal method, and ultrasonication method, with the stirring method being the most widely used.
2. The reported central anion species are generally grouped into X (halide ion), X’ (chalcogenide ion), Ox (oxoanion), POM (polyoxometalate), and H/D (hydride/deuteride).
3. The anion species affect the Ag NC volume, where POM is more effective for synthesizing larger anion-templated Ag NCs and H/D utilization is more effective for synthesizing smaller anion-templated Ag NCs.
4. When X is used as an anion species, it is possible to synthesize Ag NCs with the same number of Ag atoms but different central anions. In this case, the X–Ag and Ag–Ag distances tend to extend with the increasing size of the central X anion.
5. When X is used as the anion species, if X’ is present in the system, the cage structure can contain both Ag and X’. Because these cage structures are tough, they can also contain large X’ anions such as I^–^. Additionally, under these conditions, anion-templated Ag NCs can be synthesized in which two X anions are encapsulated within one Ag framework.
6. When X’ is used as the anion species, the cage structure can be formed with Ag only if the number of Ag atoms is small. However, under these conditions, both Ag and X’ are often included in the cage structure.
7. When Ox or POM is used as the anion species, the number of Ag atoms in the cage structure can easily be varied because of the diversity of these anions. The chemical composition and geometric structure of the resulting anion-templated Ag NCs vary depending on the number of O atoms in the central anions and the geometric structure of the central anions. Moreover, the chemical compositions of the central Ox and POM anions significantly depend on the synthesis conditions.
8. For Ag NCs using H/D as anion species, unlike other anion-templated Ag NCs, they can be synthesized not only by liquid-phase reduction but also by the reaction of X@Ag_*n*_ NCs with [BH_4_]^−^, or by the insertion of H^−^ or D^−^ into empty Ag NCs.

Considering this breadth of knowledge, many anion-templated Ag NCs with novel geometric structures may be created in the future.

## 5. Outlook

As described in this review, a large number of anion-templated Ag NCs have been reported, which indicates that the use of the anion-template method is quite effective in creating a wide variety of metal NCs. To further develop this research field, the following points should be investigated in the future:

1. **Establishment of clear synthesis design guidelines**. For the synthesis of Ag NCs with relatively simple geometric structures, such as X@Ag_8_ NCs, some reports have described synthetic design guidelines based on stoichiometry [133]. However, the syntheses of most anion-templated Ag NCs appear to be a matter of coincidence. Future research is expected to be conducted on the formation mechanism so that anion-templated Ag NCs with the desired chemical compositions and geometric structures can be synthesized.
2. **Development of applied research**. Although it was not addressed in this review, it is possible to add new functions to anion-templated Ag NCs using functional anions. However, there is currently little research on the application of anion-templated Ag NCs. This may be because of the low stability of anion-templated Ag NCs. As discussed in previous studies on SR-protected metal NCs, the stability of metal NCs can be overcome to some extent by selecting the ligand functional group [248–250]. Future studies are expected to establish methods of improving the stability of anion-templated Ag NCs and provide insights into their practical application.
3. **Utilization of other metal elements**. This review describes the current status in the field of anion-templated Ag NCs, but more research is expected to conducted on anion-templated NCs with metal elements other than Ag. Because there have been reports of anion-templated Cu NCs [251–254] and anion-templated lanthanide NCs [255–258,260], it is likely that the anion-template method can be used for the synthesis of other metal NCs. Considering the bonding between the anion species and the cage metal, the use of Ag facilitates the synthesis^259^, but more active use of other metal elements is necessary to increase the diversity of their functions and applications.
